# Comparative efficacy and safety of topical therapies for ocular involvement in Sjögren’s syndrome: a systematic review and network meta-analysis

**DOI:** 10.3389/fimmu.2026.1899484

**Published:** 2026-08-26

**Authors:** Ao Gao, Tianhua Xie, Wenrui Huang, Dan Jiang, Yunfeng Hu

**Affiliations:** 1 Department of Dermatology, The First Affiliated Hospital of Jinan University, Guangdong, China; 2 Department of Gynecology, The Fourth Clinical Medical College of Guangzhou University of Chinese Medicine, Shenzhen, Guangdong, China; 3 Department of Gynecology, Shenzhen Traditional Chinese Medicine Hospital, Shenzhen, Guangdong, China; 4 Department of Nephrology, People’s Hospital of Longhua, Shenzhen, China

**Keywords:** autologous serum, cyclosporine A, dry eye disease, network meta-analysis, platelet-rich plasma, randomized controlled trial, Sjögren’s syndrome, topical ocular therapy

## Abstract

**Objective:**

To compare the efficacy and safety of topical ocular therapies for Sjögren’s syndrome–related dry eye disease.

**Methods:**

PubMed, Embase, Web of Science, Cochrane Central, CNKI, Wanfang, VIP, and CBM were searched from inception to April 29, 2026. Randomized controlled trials of topical ocular therapies were included. Two reviewers independently screened studies, extracted data, and assessed risk of bias using RoB 2.0. A frequentist network meta-analysis was conducted in Stata 17.0. Treatment ranking was estimated using SUCRA.

**Results:**

Twelve RCTs were included. For ocular staining score, platelet-rich plasma eye drops showed a favorable effect compared with control (SMD –6.96 [95% CI –9.76, –4.16]), and autologous serum also demonstrated a favorable effect. For tear break-up time, autologous serum and platelet-rich plasma showed relatively favorable improvements compared with control (MD 3.90 [95% CI 2.24, 5.56] and MD 3.85 [95% CI 1.48, 6.22], respectively). For Schirmer test, cyclosporine A was associated with improved tear secretion compared with control (MD 5.29 [95% CI 3.80, 6.78]). For Ocular Surface Disease Index, cyclosporine A plus diquafosol sodium showed improvement compared with control (MD –2.40 [95% CI –3.21, –1.59]). Tacrolimus was associated with a higher risk of adverse events than control (OR 2.12 [95% CI 1.01, 4.42]). The certainty of evidence was generally low to moderate, and these findings should be interpreted cautiously because of sparse networks, limited direct comparisons, and uncertainty in treatment ranking.

**Conclusion:**

Topical ocular therapies showed outcome-specific advantages. Platelet-rich plasma, autologous serum, and cyclosporine A may benefit ocular surface damage, tear film stability, and tear secretion, respectively. However, sparse networks, local inconsistency, and incomplete safety reporting warrant cautious interpretation.

**Systematic review registration:**

https://www.crd.york.ac.uk/PROSPERO/, identifier CRD420261379380.

## 1 Introduction

Sjögren’s syndrome is a chronic systemic autoimmune disease characterized by immune-mediated dysfunction of the exocrine glands, particularly the lacrimal and salivary glands (1). It may occur as primary Sjögren’s syndrome or as secondary disease associated with other autoimmune disorders, including rheumatoid arthritis and systemic lupus erythematosus (2). Primary Sjögren’s syndrome affects approximately 0.01%–0.05% of the general population and predominantly occurs in women (2). Ocular involvement represents an important component of disease burden in Sjögren’s syndrome. Greenan et al. reported that ocular surface parameters were associated with vision-related quality of life in patients with primary Sjögren’s syndrome, highlighting the clinical relevance of ocular manifestations beyond subjective symptom assessment alone (3). Sjögren’s syndrome–related dry eye disease results from immune-mediated lacrimal gland damage and reduced tear secretion, leading to tear film instability, ocular surface inflammation, epithelial injury, discomfort, and visual disturbance (4). As it is often more severe and more inflammation-driven than non-Sjögren dry eye disease, appropriate topical treatment is particularly important when lubricants alone fail to control symptoms or ocular surface damage (5).

Topical ocular therapies are widely used for Sjögren’s syndrome–related dry eye disease. Artificial tears and lubricants are used to supplement tear deficiency; topical corticosteroids, cyclosporine A, and tacrolimus target ocular surface inflammation; and autologous serum and platelet-rich plasma eye drops provide growth factors and anti-inflammatory components that may support epithelial repair (4, 6, 7). However, current guidelines offer limited help in choosing among these treatments. The EULAR recommendations list artificial tears, topical anti-inflammatory agents, and serum eye drops for ocular dryness, but the treatment approach is mainly severity-based and symptomatic rather than grounded in head-to-head comparisons or formal treatment ranking (8). EULAR also notes that treatment decisions in Sjögren’s syndrome remain difficult because available studies provide insufficient information on differences in efficacy and safety among therapeutic options (8). Similarly, the TFOS DEWS III Management and Therapy Report provides an updated staged management approach for dry eye disease, emphasizing individualized treatment selection according to disease subtype, severity, and underlying mechanisms (9). However, current recommendations still provide limited guidance regarding the comparative selection of specific topical therapies, such as cyclosporine A, tacrolimus, and serum-derived eye drops, for Sjögren’s syndrome–related dry eye disease.

Thus, existing guidance supports staged escalation, but does not clearly indicate how to choose among topical medications, such as cyclosporine A, tacrolimus, and serum eye drops, for Sjögren’s syndrome–related dry eye.

Sjögren’s syndrome–related dry eye disease (SSDE) has distinct clinical and pathophysiological characteristics compared with non-Sjögren dry eye disease, primarily involving autoimmune-mediated lacrimal gland dysfunction, chronic ocular surface inflammation, and impaired tear production. Previous reviews have highlighted that SSDE may be more challenging to manage than non-Sjögren dry eye disease, and currently available therapies, including artificial tears and topical anti-inflammatory agents, often provide incomplete symptom relief and limited clinical efficacy (4). However, the comparative effectiveness of different topical therapies for SSDE remains uncertain. Previous evidence syntheses have mainly focused on general dry eye populations rather than Sjögren’s syndrome–specific populations. Tong et al. (10) evaluated topical pharmacological treatments for dry eye disease but did not specifically address SSDE or compare biological tear substitutes with anti-inflammatory therapies. Similarly, Jongkhajornpong et al. (11) performed a network meta-analysis of biological tear substitutes and topical secretagogues for dry eye disease; however, the analysis was not designed specifically for SSDE and did not include several commonly used anti-inflammatory ophthalmic treatments (12).

To our knowledge, no network meta-analysis has comprehensively compared and ranked the commonly used topical therapies for Sjögren’s syndrome–related dry eye disease. We therefore conducted a systematic review and network meta-analysis to compare the relative efficacy and safety of available topical ocular therapies for Sjögren’s syndrome–related dry eye disease and to evaluate whether different interventions demonstrate outcome-specific advantages in improving ocular surface damage, tear film stability, tear secretion, subjective symptoms, and treatment tolerability.

## 2 Methods

The present systematic review and network meta-analysis was conducted in accordance with the Preferred Reporting Items for Systematic Reviews and Meta-Analyses 2020 statement (13) and the extension statement for network meta-analyses (14). The full PRISMA-NMA checklist is provided in **Supplementary Appendix 1**. The protocol was registered in PROSPERO with the registration number CRD420261379380.

### 2.1 Search strategy

A comprehensive literature search was performed in PubMed, Embase, Web of Science, the Cochrane Central Register of Controlled Trials, China National Knowledge Infrastructure, Wanfang Data, VIP Database, and China Biology Medicine Database. Each database was searched from inception to April 29, 2026. The search strategy was developed according to the PICOS framework by combining subject headings and free-text terms related to “Sjögren’s syndrome, “ “dry eye, “ “keratoconjunctivitis sicca, “ “artificial tears, “ “cyclosporine, “ “tacrolimus, “ “corticosteroid, “ “autologous serum, “ “platelet-rich plasma, “ and “randomized controlled trial.” Search terms were adapted for both English and Chinese databases. In addition, ClinicalTrials.gov and the reference lists of included studies and relevant reviews were manually searched to identify additional eligible trials. The detailed search strategy is provided in **Supplementary Appendix 2**.

The search strategy was developed to identify randomized controlled trials evaluating the comparative efficacy and safety of different topical ocular therapies for Sjögren’s syndrome–related dry eye disease.

### 2.2 Eligibility criteria

#### 2.2.1 Population

Patients with Sjögren’s syndrome–related dry eye disease, including primary or secondary Sjögren’s syndrome (15), were eligible. Both Sjögren’s syndrome and dry eye disease had to be diagnosed using recognized or clearly reported criteria in the original studies. Sjögren’s syndrome was required to be diagnosed according to established classification criteria, including the 2002 American–European Consensus Group criteria, the 2012 SICCA/ACR criteria, the 2016 ACR/EULAR criteria, or other clearly stated diagnostic criteria. Dry eye disease was defined based on ocular symptoms (such as dryness, foreign body sensation, burning, or ocular discomfort) combined with objective ocular surface assessments, including tear breakup time, Schirmer test, ocular surface staining, tear meniscus height, or tear osmolarity (16).Studies with mixed dry eye populations were included only if Sjögren’s syndrome–related dry eye data were separately extractable No restrictions were imposed on sex, race, ethnicity, age, or disease duration.

#### 2.2.2 Intervention and comparison

Eligible interventions were topical ocular therapies evaluated for Sjögren’s syndrome–related dry eye disease, including cyclosporine A, tacrolimus, topical corticosteroids, autologous serum eye drops, platelet-rich plasma eye drops, and other topical ocular medications evaluated in eligible randomized controlled trials (9). Eligible comparators included placebo, vehicle, blank control, artificial tears, sodium hyaluronate, standard care, or other topical ocular medications. For multi-arm trials, only treatment arms relevant to this network meta-analysis were included. Studies evaluating only local non-pharmacological procedures, such as punctal occlusion or punctal plugs, were excluded.

Artificial tears, sodium hyaluronate, vehicle, placebo, blank control, and standard care were combined into a single control node, defined as CTL. These comparator arms were used as inactive, vehicle-matched, or supportive treatments in the original trials, rather than as active anti-inflammatory, secretagogue, or blood-derived therapies. Artificial tears and sodium hyaluronate mainly provide lubrication or tear supplementation, whereas vehicle and placebo were used as formulation or inactive controls. Combining these arms improved network connectivity and allowed active topical therapies to be compared against a common control condition.

#### 2.2.3 Outcomes

Primary outcomes included ocular staining score, tear breakup time, Schirmer test, and adverse events. Secondary outcomes included OSDI. Safety outcomes included any adverse events, serious adverse events, and discontinuation due to adverse events, as reported in the original studies. Adverse events were extracted according to the assessment methods described in each trial, including investigator-reported events, participant-reported symptoms, or other safety monitoring procedures when available.Tear breakup time was extracted according to the definitions provided in the original studies. Although both fluorescein breakup time and non-invasive breakup time were considered eligible measurements, all included studies reporting tear breakup time in this network meta-analysis used fluorescein-based assessments. Tear osmolarity was considered as a potential objective outcome; however, it was not included in the quantitative synthesis because insufficient eligible randomized controlled trials reported this measure in patients with Sjögren’s syndrome–related dry eye disease.

#### 2.2.4 Study design

RCTs published in either domestic or international sources were considered eligible, irrespective of blinding procedures or allocation concealment. Studies were eligible regardless of publication language. Single-arm studies were excluded because they lacked comparative control groups and could not provide relative treatment effects required for network meta-analysis. Grey literature, including conference abstracts, unpublished studies, and trial registry results without sufficient peer-reviewed outcome data, was not included because the available information was insufficient for reliable risk assessment and quantitative synthesis. All included studies were required to provide sufficient data for efficacy and safety evaluation.

### 2.3 Study identification and data acquisition

All retrieved records were imported into EndNote 21 for duplicate removal. Two reviewers independently screened titles and abstracts according to the predefined eligibility criteria, and studies with uncertain eligibility were included for full-text assessment. Full texts were then reviewed to identify eligible trials, and reasons for exclusion were recorded. Any disagreements were resolved through discussion or consultation with a third reviewer (14).

Data extraction was independently conducted by two reviewers using a standardized form, with discrepancies resolved by discussion or verification by a third reviewer (14, 17, 18). Extracted data included study characteristics, such as first author, publication year, and country; participant characteristics, including sample size, age, sex, and disease duration; intervention details, including treatment type, dosage, frequency, treatment duration, comparator, and follow-up time; methodological information, including randomization, allocation concealment, blinding, and completeness of outcome data; and outcome measures, including ocular staining score, tear breakup time, Schirmer test, adverse events, and Ocular Surface Disease Index. Information on concomitant or adjunctive treatments was also extracted when reported, including artificial tears, sodium hyaluronate, standard-of-care therapy, and other supportive ocular treatments. These factors were considered during assessment of clinical heterogeneity and interpretation of treatment effects. When multiple follow-up time points were reported, data from the longest available follow-up were extracted unless otherwise specified.

### 2.4 Appraisal of bias and quality evaluation

We evaluated potential bias across all eligible studies using the Cochrane Risk of Bias 2.0 tool (19), encompassing five critical domains: randomization process, deviations from intended interventions, missing outcome data, measurement of outcomes, and selective reporting. Each domain was systematically rated according to the signaling questions outlined in the RoB 2.0 guidelines. Studies with low risk across all domains were classified as low risk of bias; those with some concerns but no high-risk judgments were rated as having some concerns; and those with at least one high-risk domain were considered high risk of bias. Two reviewers independently performed the assessments, resolving disagreements through discussion or consultation with a third reviewer when necessary.

Additionally, the certainty of evidence was evaluated using the Confidence in Network Meta-Analysis framework (20, 21), which assesses six dimensions: within-study bias, reporting bias, indirectness, imprecision, heterogeneity, and incoherence. The confidence in each comparison was appraised comprehensively based on both direct and indirect evidence across these domains.

### 2.5 Data synthesis and analysis

A frequentist network meta-analysis was conducted using Stata 17.0 to integrate direct and indirect evidence for comprehensive comparative estimates. Network plots were generated to illustrate the relationships among topical ocular medications. Effect sizes for dichotomous outcomes, including adverse events, were expressed as odds ratios (ORs) with 95% confidence intervals (CIs).

For continuous outcomes, tear breakup time, Schirmer test, and Ocular Surface Disease Index were analyzed using mean differences (MDs) with 95% CIs when the same units or scoring systems were used across studies. Ocular staining scores were analyzed using standardized mean differences (SMDs) with 95% CIs because different ocular surface staining scoring systems, including SICCA ocular staining score, Oxford score, and van Bijsterveld score, were used across the included studies. Although ocular staining scores are derived from ordinal grading systems, they were reported as numerical values with means and standard deviations in the eligible randomized controlled trials, allowing quantitative synthesis using SMDs. For other outcomes measured using different scales, SMDs were also used. Multi-arm trials evaluating different doses of the same intervention were combined into a single treatment node when appropriate.

Between-study heterogeneity was estimated using the restricted maximum likelihood method and classified by τ² values as low heterogeneity (<0.04), low-to-moderate heterogeneity (0.04–0.16), moderate-to-high heterogeneity (0.16–0.36), or high heterogeneity (>0.36). Global inconsistency was assessed using the design-by-treatment interaction model. The transitivity assumption was evaluated by descriptively comparing potential effect modifiers across included studies, including Sjögren’s syndrome diagnostic criteria, disease subtype (primary or secondary Sjögren’s syndrome), baseline dry eye severity, concomitant treatments, treatment duration, follow-up duration, and outcome measurement scales. The detailed assessment of these potential effect modifiers is presented in **Supplementary Table 2**.

Treatment rankings were visualized using cumulative ranking curves and surface under the cumulative ranking curve (SUCRA) plots. SUCRA values closer to 1 indicated a higher probability of superior efficacy for efficacy outcomes and better safety for adverse events. League tables were constructed to present pairwise comparisons, statistical differences, and effect estimates between interventions.

## 3 Results

### 3.1 Study selection

**Figure 1:**
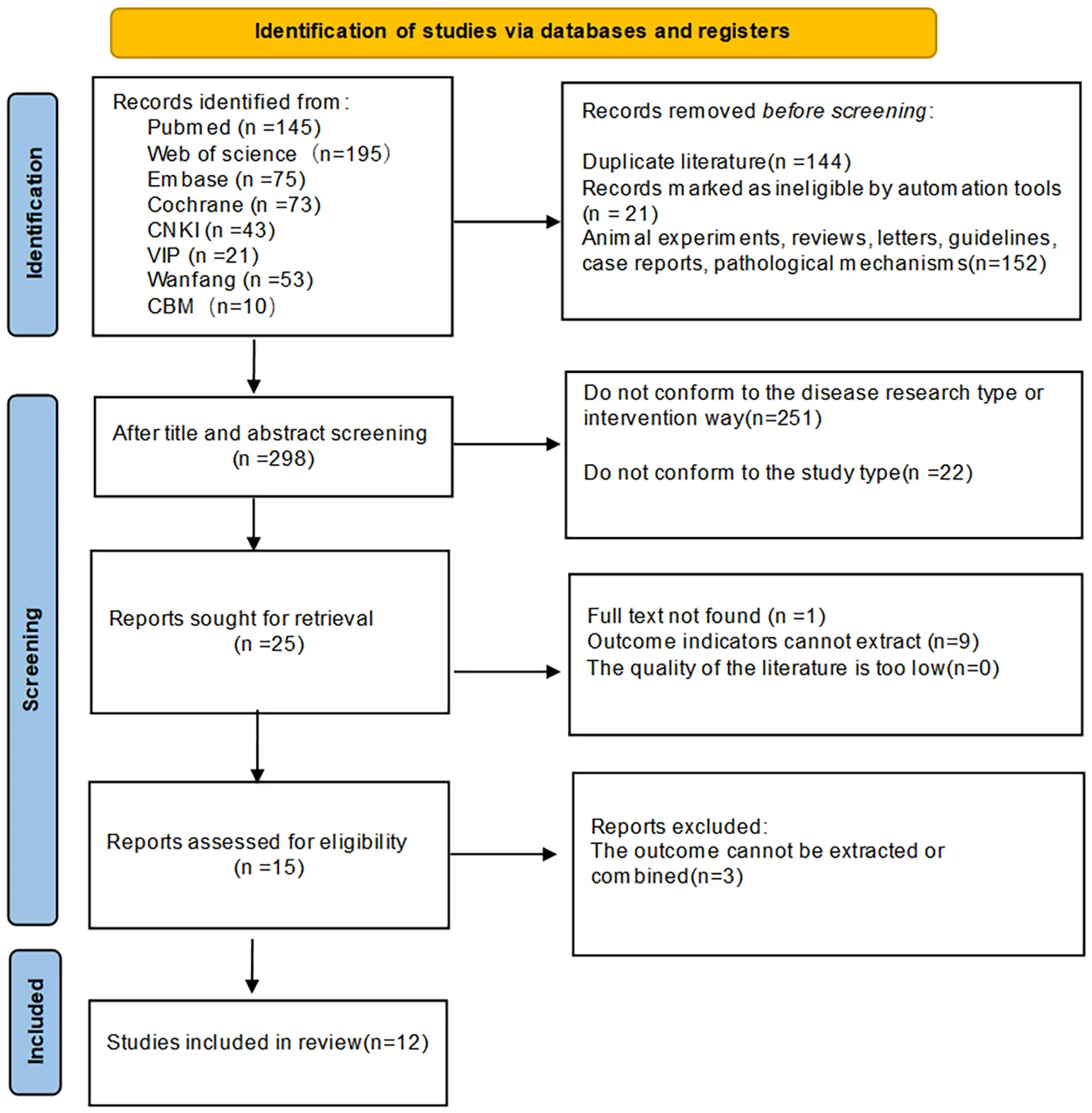
Study selection flow diagram. Flow diagram of the identification, screening, eligibility assessment, and inclusion process of randomized controlled trials evaluating topical ocular therapies for Sjögren’s syndrome–related dry eye disease according to the PRISMA guidelines.

A total of 615 records were identified through database searches: PubMed (n = 145), Web of Science (n = 195), Embase (n = 75), Cochrane Library (n = 73), CNKI (n = 43), VIP (n = 21), Wanfang (n = 53), and CBM (n = 10). After removing duplicates (n = 144), records marked as ineligible by automation tools (n = 21), and records involving animal studies, reviews, letters, guidelines, case reports, or mechanistic studies (n = 152), 298 records remained for title and abstract screening. Of these, 251 records were excluded because they did not meet the disease or intervention criteria, and 22 were excluded because they did not meet the study design criteria. Twenty-five reports were sought for full-text assessment. One report was excluded because the full text was unavailable. Among the remaining full-text reports, nine were excluded because outcome data could not be extracted, and three were excluded because outcomes could not be extracted or combined. Finally, 12 RCTs were included in the network meta-analysis. The study selection process is shown in **Figure 1**.

### 3.2 Characteristics of included studies

The 12 included RCTs involved patients with Sjögren’s syndrome–related dry eye disease. The interventions included topical cyclosporine A (CsA), topical tacrolimus (TAC), topical corticosteroids (ST), autologous serum eye drops (AS), platelet-rich plasma eye drops (PRP), Lacripep eye drops (LAC), topical diquafosol sodium (DQS), cyclosporine A combined with diquafosol sodium (CsA+DQS), and control treatment (CTL). The control group included artificial tears, lubricant eye drops, vehicle, placebo, polyvinylpyrrolidone, sodium hyaluronate, or background control as defined in the original trials. The primary outcomes were ocular staining score (OSS), tear break-up time (TBUT), Schirmer test, and adverse events (AEs). The Ocular Surface Disease Index (OSDI) was analyzed as a secondary outcome. Detailed characteristics of the included studies are shown in **Appendix 3** and **Supplementary Table 3.1**.

### 3.3 Risk of bias, certainty of evidence, and consistency

Risk of bias was assessed using the RoB 2 tool. Among the 12 included RCTs, 2 studies were judged as having a low overall risk of bias, 8 raised some concerns, and 2 had a high risk of bias. All studies were rated as low risk in the randomization process. The main concerns were related to deviations from intended interventions, missing outcome data, measurement of outcomes, and selection of the reported result (22). Details are provided in **Appendix 4**.

Consistency assessments showed no significant global inconsistency across the networks (**Appendix 5**; **Supplementary Table 5.1**). The τ² values were all below 0.4, indicating that no high heterogeneity was detected across the networks. Node-splitting analysis showed no local inconsistency for most comparisons, although potential local inconsistency was observed in the TBUT network for CTL versus CsA, CTL versus TAC, and CsA versus TAC (**Appendix 5**; **Supplementary Table 5.2**). The certainty of evidence assessed using the CINeMA framework was generally rated as low to moderate across most outcomes (**Appendix 8**). Funnel plots showed no clear evidence of asymmetry (**Appendix 9**). However, the interpretation of publication bias assessment was limited by the small number of included studies and the sparse network structure (23).

### 3.4 Ocular staining score

**Figure 2:**
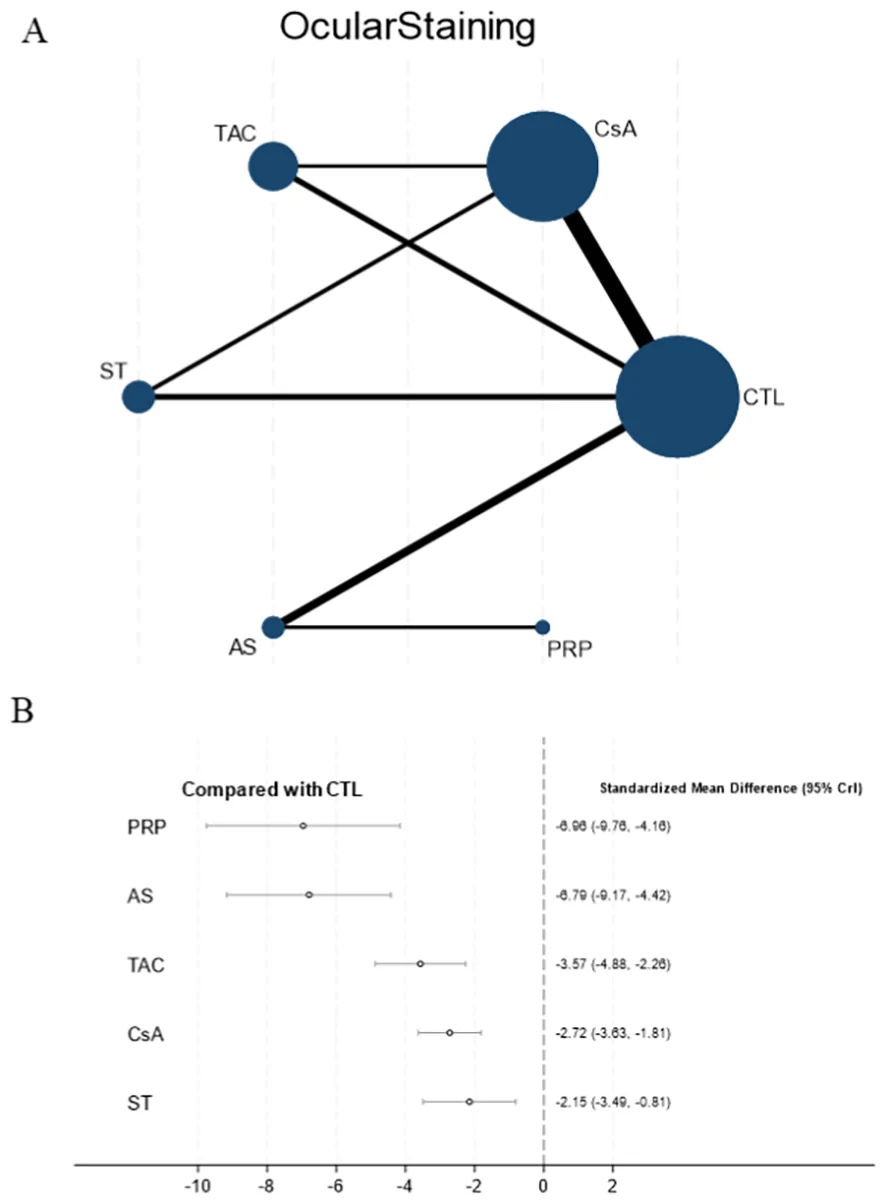
Network meta-analysis of ocular staining score. **(A)** Network plot showing direct comparisons among topical ocular therapies. The size of each node represents the number of participants receiving each intervention, and the thickness of connecting lines represents the number of direct comparisons. **(B)** Forest plot showing standardized mean differences (SMDs) with 95% confidence intervals (CIs) for ocular staining score compared with control treatment. CTL, control treatment; CsA, cyclosporine A; TAC, tacrolimus; ST, topical corticosteroids; AS, autologous serum eye drops; PRP, platelet-rich plasma eye drops.

This NMA evaluated OSS in 9 RCTs involving 421 participants. As shown in **Figure 2A**, the network included six treatment nodes: CTL, CsA, TAC, ST, AS, and PRP. The forest plot showed that all active interventions significantly reduced OSS compared with CTL (**Figure 2B**). PRP showed the largest reduction in OSS (SMD = −6.96, 95% CI −9.76 to −4.16; SUCRA = 91.6%), followed by AS (SMD = −6.79, 95% CI −9.17 to −4.42; SUCRA = 87.9%), TAC (SMD = −3.57, 95% CI −4.88 to −2.26), CsA (SMD = −2.72, 95% CI −3.63 to −1.81), and ST (SMD = −2.15, 95% CI −3.49 to −0.81).

Further pairwise comparisons are provided in **Appendix 7**; **Supplementary Table 7.1**. League-table comparisons showed that AS was superior to CsA (SMD = 4.08, 95% CI 1.53 to 6.62) and ST (SMD = 4.64, 95% CI 1.91 to 7.37). PRP was also superior to CsA (SMD = 4.24, 95% CI 1.30 to 7.19) and ST (SMD = 4.81, 95% CI 1.71 to 7.92). According to SUCRA values, PRP showed the highest probability of ranking for OSS improvement, followed by AS, TAC, CsA, ST, and CTL. According to CINeMA, the overall certainty of evidence for OSS was mainly rated as low to moderate (**Appendix 8**; **Supplementary Table 8.1**).

Because OSS represents a composite measure of ocular surface staining, separately reported corneal and conjunctival staining outcomes were additionally explored. These outcomes were not quantitatively synthesized due to limited reporting, heterogeneous staining assessment methods, and insufficient comparable treatment networks.Four RCTs reported separate corneal and/or conjunctival staining outcomes. Kang et al. (6) reported improvements in both corneal and conjunctival staining outcomes following PRP and AS treatment. Bachtalia et al. (24) observed improvements in corneal fluorescein staining and conjunctival lissamine green staining after AS treatment. Moscovici et al. (25) reported reduced fluorescein and Rose Bengal staining scores following topical tacrolimus treatment. Aragona et al. (26) reported improvements in corneal fluorescein staining and conjunctival lissamine green staining after topical corticosteroid therapy.

### 3.5 Tear break-up time

**Figure 3:**
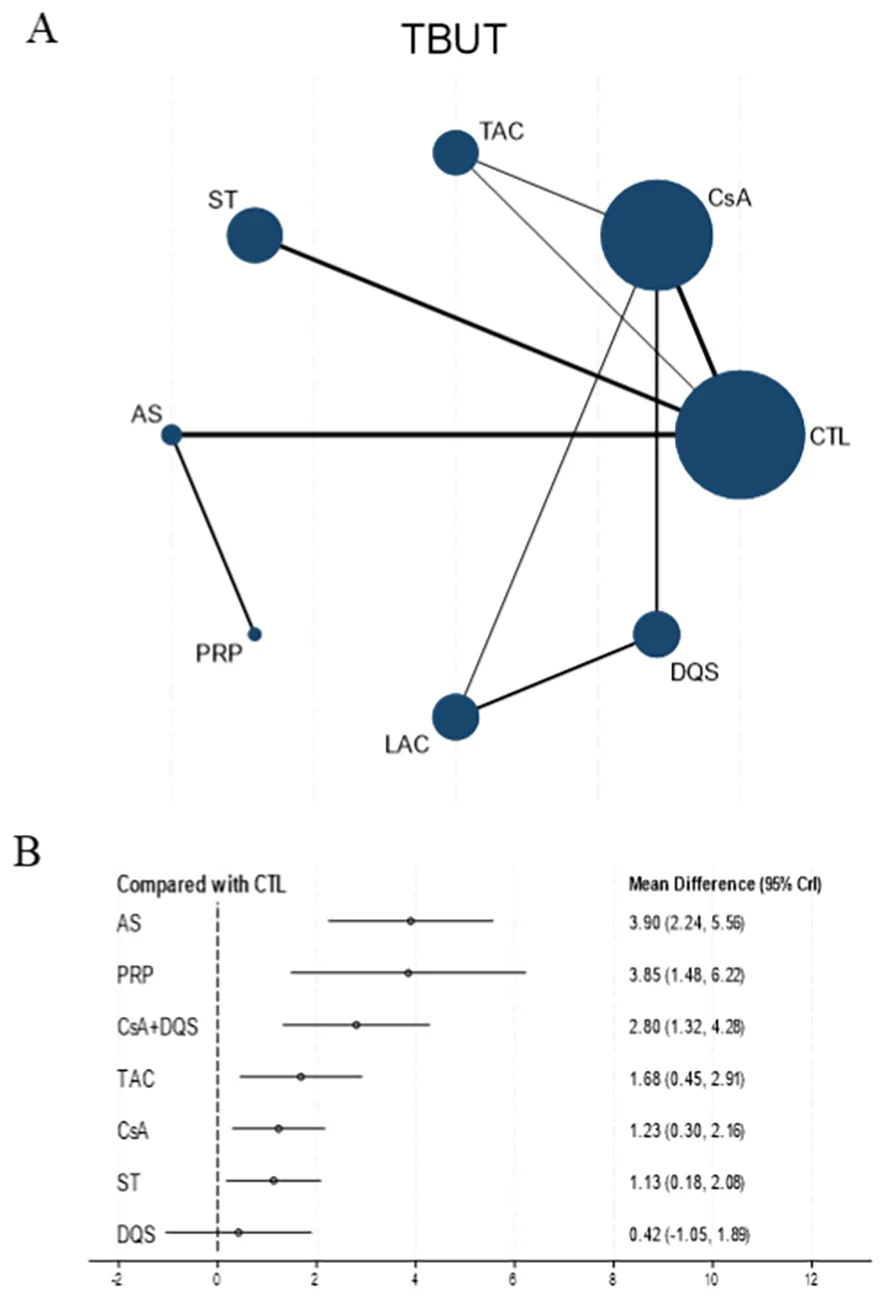
Network meta-analysis of tear break-up time. **(A)** Network plot showing direct comparisons among topical ocular therapies. The size of each node represents the number of participants receiving each intervention, and the thickness of connecting lines represents the number of direct comparisons. **(B)** Forest plot showing mean differences (MDs) with 95% confidence intervals (CIs) for tear break-up time compared with control treatment. CTL, control treatment; CsA, cyclosporine A; TAC, tacrolimus; ST, topical corticosteroids; AS, autologous serum eye drops; PRP, platelet-rich plasma eye drops; DQS, diquafosol sodium; LAC, Lacripep eye drops.

This NMA evaluated TBUT in 9 RCTs involving 520 participants. As shown in **Figure 3A**, the network included CTL, CsA, TAC, ST, AS, PRP, LAC, DQS, and CsA+DQS. The forest plot showed that AS produced the greatest improvement in TBUT compared with CTL (MD = 3.90, 95% CI 2.24 to 5.56; SUCRA = 90.5%), followed by PRP (MD = 3.85, 95% CI 1.48 to 6.22; SUCRA = 87.2%), CsA+DQS (MD = 2.80, 95% CI 1.32 to 4.28; SUCRA = 75.6%), TAC (MD = 1.68, 95% CI 0.45 to 2.91), CsA (MD = 1.23, 95% CI 0.30 to 2.16), and ST (MD = 1.13, 95% CI 0.18 to 2.08). DQS did not show a statistically significant difference compared with CTL (MD = 0.42, 95% CI −1.05 to 1.89) (**Figure 3B**).

Further pairwise comparisons are detailed in **Appendix 7**, **Supplementary Table 7.2**. League-table comparisons showed that AS was superior to CsA (MD = 2.67, 95% CI 0.76 to 4.58), TAC (MD = 2.22, 95% CI 0.18 to 4.26), and ST (MD = 2.77, 95% CI 0.85 to 4.69). PRP was superior to CsA (MD = 2.62, 95% CI 0.06 to 5.18) and ST (MD = 2.72, 95% CI 0.16 to 5.29). CsA+DQS was superior to DQS (MD = 2.38, 95% CI 0.76 to 4.00). According to SUCRA values, AS showed the highest probability of ranking for TBUT improvement, followed by PRP and CsA+DQS. According to CINeMA, the overall certainty of evidence for TBUT was rated as low to moderate (**Appendix 8**; **Supplementary Table 8.2**). Local inconsistency was detected in comparisons involving CTL, CsA, and TAC. Sensitivity analysis excluding studies judged as having a high risk of bias did not substantially alter the direction of treatment effects (**Appendix 11**; **Supplementary Table 11.1**).

### 3.6 Schirmer test

**Figure 4:**
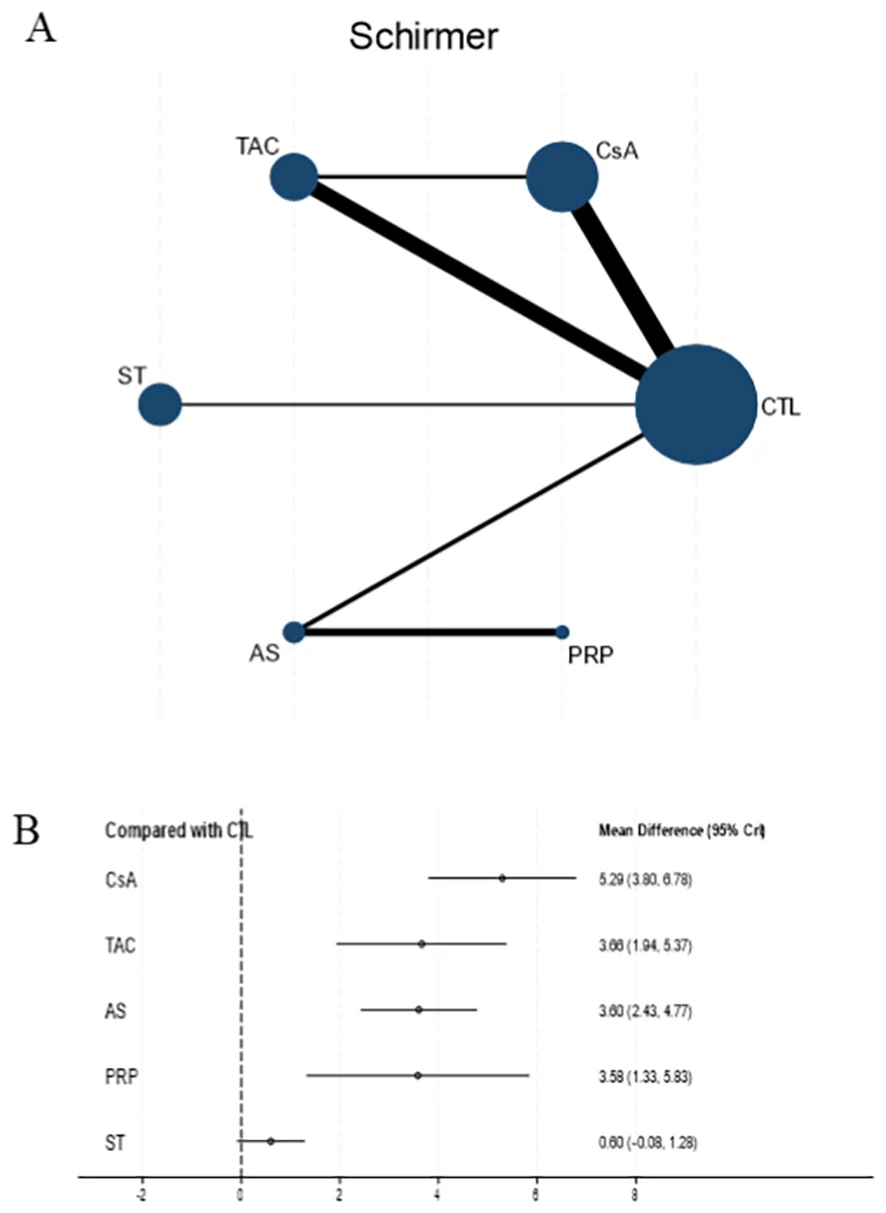
Network meta-analysis of Schirmer test. **(A)** Network plot showing direct comparisons among topical ocular therapies. The size of each node represents the number of participants receiving each intervention, and the thickness of connecting lines represents the number of direct comparisons. **(B)** Forest plot showing mean differences (MDs) with 95% confidence intervals (CIs) for Schirmer test values compared with control treatment. CTL, control treatment; CsA, cyclosporine A; TAC, tacrolimus; ST, topical corticosteroids; AS, autologous serum eye drops; PRP, platelet-rich plasma eye drops.

The NMA for Schirmer test included 7 RCTs involving 390 participants. As shown in **Figure 4A**, the network included CTL, CsA, TAC, ST, AS, and PRP. Compared with CTL, CsA showed the greatest improvement in Schirmer test values (MD = 5.29, 95% CI 3.80 to 6.78; SUCRA = 97.1%), followed by TAC (MD = 3.66, 95% CI 1.94 to 5.37), AS (MD = 3.60, 95% CI 2.43 to 4.77), and PRP (MD = 3.58, 95% CI 1.33 to 5.83). ST did not show a statistically significant difference compared with CTL (MD = 0.60, 95% CI −0.08 to 1.28) (**Figure 4B**).

Further pairwise comparisons are presented in **Appendix 7**, **Supplementary Table 7.3**. According to SUCRA values, CsA showed the highest probability of ranking for improving Schirmer test values. According to CINeMA, the overall certainty of evidence for Schirmer test was mainly rated as low to moderate (**Appendix 8**; **Supplementary Table 8.3**).

### 3.7 Adverse events

**Figure 5:**
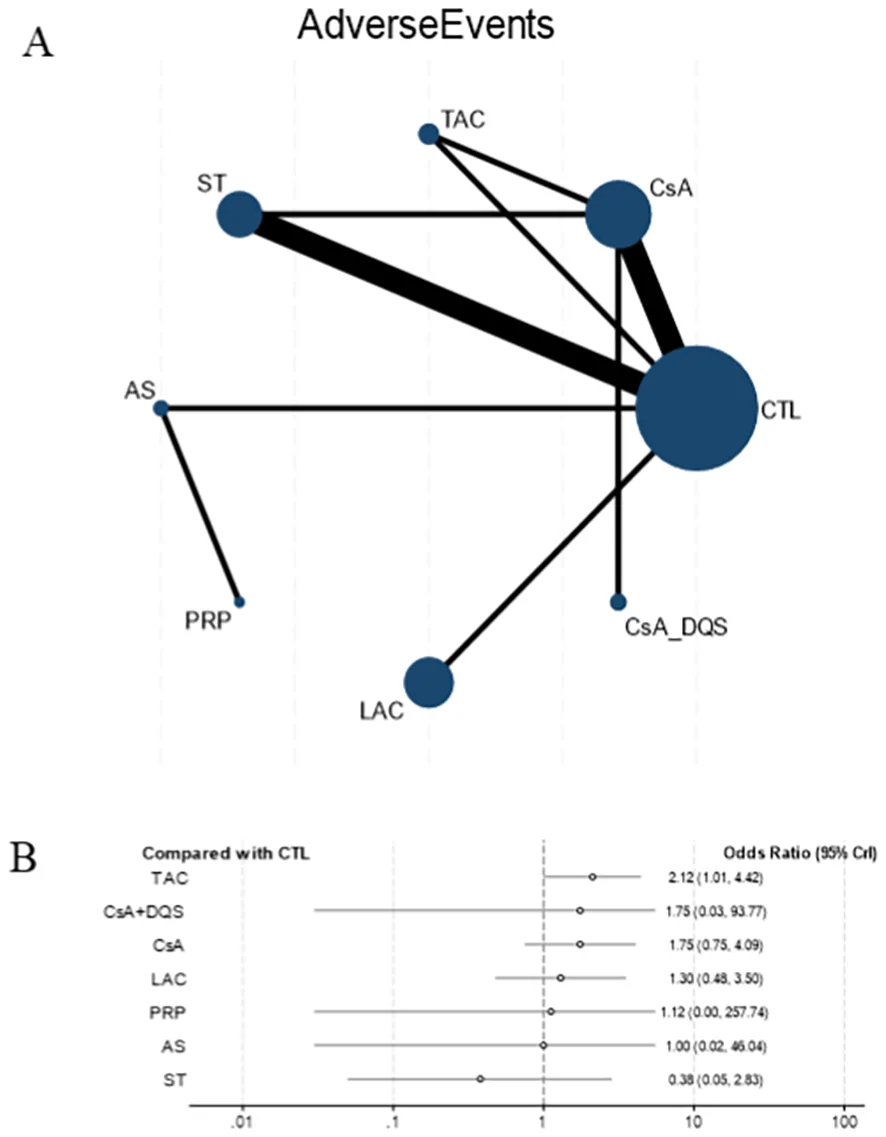
Network meta-analysis of adverse events. **(A)** Network plot showing direct comparisons among topical ocular therapies. The size of each node represents the number of participants receiving each intervention, and the thickness of connecting lines represents the number of direct comparisons. **(B)** Forest plot showing odds ratios (ORs) with 95% confidence intervals (CIs) for adverse events compared with control treatment. CTL, control treatment; CsA, cyclosporine A; TAC, tacrolimus; ST, topical corticosteroids; AS, autologous serum eye drops; PRP, platelet-rich plasma eye drops; DQS, diquafosol sodium; LAC, Lacripep eye drops.

The NMA on AEs included 11 RCTs involving 697 participants. As shown in **Figure 5A**, the network included CTL, CsA, TAC, ST, AS, PRP, LAC, and CsA+DQS. Compared with CTL, TAC was associated with a significantly higher risk of AEs (OR = 2.12, 95% CI 1.01 to 4.42). CsA+DQS (OR = 1.75, 95% CI 0.03 to 93.77), CsA (OR = 1.75, 95% CI 0.75 to 4.09), LAC (OR = 1.30, 95% CI 0.48 to 3.50), PRP (OR = 1.12, 95% CI 0.00 to 257.74), AS (OR = 1.00, 95% CI 0.02 to 46.04), and ST (OR = 0.38, 95% CI 0.05 to 2.83) did not differ significantly from CTL (**Figure 5B**).

Further comparisons are shown in **Appendix 7**, **Supplementary Table 7.4**. League-table comparisons also indicated a higher AE risk for TAC than CTL (OR = 2.12, 95% CI 1.01 to 4.42), while no other statistically significant differences were observed among treatments.

According to SUCRA values, ST showed the highest probability of ranking for fewer reported adverse events. This finding should not be interpreted as evidence of superior long-term safety because adverse-event reporting was limited. However, several AE comparisons had wide 95% CIs, so this ranking should be interpreted cautiously and should not be taken as evidence of long-term safety. According to CINeMA, the overall certainty of evidence for AEs was mainly rated as low, with the CTL versus TAC comparison rated as moderate (**Appendix 8**; **Supplementary Table 8.4**).

### 3.8 Secondary outcome: ocular surface disease index

OSDI was analyzed as a secondary outcome, including 3 RCTs with 254 participants. Compared with CTL, CsA (MD = −1.78, 95% CI −2.41 to −1.16), ST (MD = −1.06, 95% CI −1.48 to −0.65), DQS (MD = −1.68, 95% CI −2.48 to −0.88), and CsA+DQS (MD = −2.40, 95% CI −3.21 to −1.59) significantly improved OSDI. Further comparisons showed that CsA+DQS was superior to CsA alone (MD = −0.62, 95% CI −1.14 to −0.10). No other statistically significant differences were observed among active interventions.

### 3.9 Sensitivity analysis

Sensitivity analyses were performed to assess the impact of different control-node definitions on the robustness of the network meta-analysis results. Because the primary analysis combined various control interventions, including inactive controls and supportive ocular surface treatments, an alternative control definition was explored by restricting the CTL node to inactive comparators (placebo or vehicle controls). Only two studies Moscovici et al. (25) and Tauber et al. (26)] used purely inactive comparators; therefore, this sensitivity analysis was considered exploratory.

The results of sensitivity analyses for OSS, TBUT, Schirmer test, and adverse events were generally consistent with the primary analyses, with no substantial changes in the direction of treatment effects (**Appendix 11**).

## 4 Discussion

### 4.1 Principal findings

In this network meta-analysis of 12 randomized controlled trials, we compared the efficacy and safety of topical ocular therapies for Sjögren’s syndrome–related dry eye disease. The interventions evaluated included cyclosporine A (CsA), tacrolimus (TAC), topical corticosteroids (ST), autologous serum eye drops (AS), platelet-rich plasma eye drops (PRP), Lacripep eye drops (LAC), diquafosol sodium (DQS), CsA combined with DQS, and control treatment (CTL). The main outcomes were ocular staining score (OSS), tear break-up time (TBUT), Schirmer test, and adverse events (AEs), with the Ocular Surface Disease Index (OSDI) analyzed as a secondary outcome. PRP and AS showed relatively favorable effects on ocular staining outcomes, whereas AS and PRP demonstrated greater improvements in tear film stability outcomes. CsA showed the greatest improvement in Schirmer test values compared with CTL. However, these findings should be interpreted cautiously because treatment rankings were derived from a sparse evidence network, and several comparisons relied mainly on indirect evidence. For safety outcomes, ST showed a favorable short-term ranking profile; however, this finding should be interpreted cautiously because safety evidence was limited and several estimates had wide confidence intervals.

For OSDI, CsA+DQS showed the largest improvement. Overall, these findings suggest that topical ocular therapies may show different advantages across ocular surface damage, tear film stability, tear secretion, subjective symptoms, and safety.

### 4.2 Comparisons with other studies

Earlier guidelines and reviews have supported stepwise and individualized management for dry eye disease, but they have not provided a clear comparative ranking of topical therapies specifically for Sjögren’s syndrome–related dry eye disease. Jones et al. summarized treatment options in the TFOS DEWS II Management and Therapy Report, including tear supplementation, anti-inflammatory therapy, tear conservation, and biological tear substitutes (9). Ramos-Casals et al. proposed EULAR recommendations for Sjögren’s syndrome and included topical ocular therapies as symptomatic treatment for ocular dryness (8). These recommendations support the general use of topical therapy, whereas the present NMA further compares multiple topical options within the same analytical framework.

Previous studies on AS and PRP mainly support their role in ocular surface repair, but most evidence comes from single studies, small randomized trials, or direct comparisons between blood-derived preparations. Tsubota et al. reported that AS improved ocular surface vital staining in patients with Sjögren’s syndrome–related dry eye disease (27). Kang et al. compared PRP with AS in primary Sjögren’s syndrome dry eye and found that both treatments improved ocular surface staining and TBUT, without a clear between-group difference at the main follow-up points (6). Avila et al. also reported improvement in tear-related and subjective parameters after PRP treatment in severe dry eye (28). These studies support the direction of our findings, but they do not compare AS or PRP with anti-inflammatory agents, DQS, Lacripep eye drops, or combination therapy in a unified network. Our NMA adds this comparative layer and suggests that PRP and AS may be relatively more favorable for OSS, while AS and PRP also ranked higher for TBUT. Evidence from reviews on plasma-derived ocular surface treatments and blood component therapy also supports the biological plausibility of these findings, although direct comparative certainty remains limited (29, 30).

Evidence for CsA and TAC has mainly focused on anti-inflammatory therapy. Sall et al. and Stevenson et al. reported that cyclosporine ophthalmic emulsion improved signs and symptoms in moderate-to-severe dry eye disease (31, 32). Moscovici et al. evaluated 0.03% TAC eye drops in Sjögren’s syndrome–related dry eye disease and reported improvement in ocular surface parameters (25). Moawad et al. compared TAC 0.03% with CsA 0.05% in Sjögren’s syndrome–related dry eye disease and found improvement with both treatments (33). These studies suggest that calcineurin inhibitors may have therapeutic value, but they do not clarify how CsA or TAC compares with biological tear substitutes, secretagogues, Lacripep eye drops, or combination regimens across different outcomes. In our analysis, CsA ranked highest for Schirmer test, whereas TAC was associated with a higher risk of AEs. This supports a distinction between efficacy and tolerability when interpreting calcineurin inhibitor therapy.

The finding for CsA+DQS also extends prior evidence. DQS is a P2Y2 receptor agonist that can promote tear fluid and mucin secretion (34–36). Kinoshita et al. reported that DQS ophthalmic solution was effective and safe for dry eye syndrome in a randomized phase 2 trial (34), and Yokoi et al. showed that 3% DQS increased tear fluid volume on the ocular surface (35). In this NMA, CsA+DQS ranked favorably for TBUT and showed the largest improvement in OSDI. This may reflect combined anti-inflammatory and secretagogue effects, but the number of contributing studies was limited. Therefore, this result should be viewed as suggestive rather than definitive.

Taken together, the value of this study is not in re-establishing that topical ocular therapies can improve dry eye outcomes. Its main contribution is the comparison of relative advantages across OSS, TBUT, Schirmer test, OSDI, and AEs. The findings indicate an outcome-specific pattern: PRP and AS ranked higher for ocular surface damage, AS and PRP ranked higher for tear film stability, CsA ranked highest for tear secretion, and CsA+DQS showed the largest improvement in subjective symptoms. This pattern should be interpreted together with the certainty of evidence, network structure, and directness of comparisons.

It should be noted that topical cyclosporine A is available in different concentrations and formulations, including 0.05%, 0.09%, and 0.1% preparations with different vehicle systems. In this network meta-analysis, cyclosporine A formulations were analyzed as a single treatment node because the available randomized evidence was insufficient to support separate comparisons among different concentrations or delivery systems. Although this approach improved network connectivity, potential differences in ocular bioavailability, pharmacokinetics, tolerability, and clinical efficacy among formulations should be considered when interpreting the results.

### 4.3 Practical implications

From a clinical perspective, the findings of this network meta-analysis do not support a definitive hierarchy among topical therapies for Sjögren’s syndrome–related dry eye disease. Instead, the available evidence suggests an outcome-specific treatment pattern, indicating that different therapies may have potential advantages for distinct clinical manifestations. Platelet-rich plasma (PRP) and autologous serum (AS) eye drops showed relatively favorable effects on ocular staining outcomes, suggesting that they may be considered in patients with prominent ocular surface epithelial damage. AS and PRP also demonstrated potential benefits for tear film stability based on tear break-up time outcomes. In contrast, cyclosporine A (CsA) showed a favorable effect on Schirmer test values, suggesting a potential role in patients with predominant aqueous tear deficiency. However, these findings should not be interpreted as establishing a superior treatment hierarchy because the certainty of evidence was generally low to moderate, direct head-to-head comparisons were limited, and several treatment comparisons were derived from sparse evidence networks. Therefore, treatment selection should be individualized according to objective ocular findings, symptom burden, treatment availability, patient preference, and long-term tolerability. Furthermore, although several interventions demonstrated statistically significant improvements, the clinical interpretation of some effect estimates requires caution. The large SMDs observed for OSS, particularly for platelet-rich plasma and autologous serum, are difficult to translate into absolute clinical benefits because different ocular staining systems with varying scoring ranges were synthesized. Therefore, these findings should be interpreted as relative treatment effects rather than direct estimates of clinically observable improvement.

AS and PRP may also be useful when tear film instability is prominent, with TBUT improvements of approximately 3.90 and 3.85 seconds compared with CTL, respectively. These findings suggest improved tear film stability, although the clinical impact on visual function requires further confirmation. CsA may be more relevant when aqueous tear deficiency is the main concern, as it increased Schirmer test values by approximately 5.29 mm compared with CTL. However, TBUT rankings should be interpreted cautiously because local inconsistency was detected. In addition, improvements in Schirmer test values should be interpreted together with ocular staining outcomes and patient-reported symptoms, as increased tear secretion alone may not fully reflect restoration of ocular surface condition or improvement in disease burden.

The OSDI and safety findings should be interpreted more cautiously. Although CsA+DQS showed the largest OSDI improvement, the absolute effect was relatively small and was based on only three RCTs; therefore, the statistically significant result may not necessarily represent a clearly patient-perceptible benefit. For safety, ST ranked highest only for fewer short-term reported AEs and should not be interpreted as evidence of long-term corticosteroid safety. TAC was associated with a higher risk of AEs than CTL, suggesting that local tolerability should be considered when selecting calcineurin inhibitors.

These findings suggest an outcome-specific treatment pattern: PRP and AS may be more suitable when ocular surface epithelial damage is the main concern, AS and PRP may be useful when tear film instability predominates, and CsA may be more appropriate when tear secretion is markedly reduced. However, TBUT rankings should be considered suggestive because local inconsistency was detected, and treatment choice should integrate symptom burden, objective ocular findings, treatment availability, preparation standards for blood-derived eye drops, patient preference, and tolerability rather than relying on rankings alone.

### 4.4 Limitations, and future directions

Several limitations should be acknowledged. First, only 12 RCTs were included, and several treatment nodes were supported by a limited number of trials. Direct head-to-head comparisons between active treatments were scarce, and some comparisons relied mainly on indirect evidence; therefore, treatment rankings should be interpreted as comparative signals rather than definitive evidence of superiority. Second, heterogeneity existed in treatment regimens, baseline disease severity, follow-up duration, and outcome assessment methods. Different control interventions, including artificial tears, sodium hyaluronate, vehicle, placebo, polyvinylpyrrolidone, and background treatments, were merged into a single CTL node to improve network connectivity, although clinical differences among these controls may have contributed to heterogeneity. In addition, OSS was derived from ordinal grading systems rather than truly continuous measurements; although SMDs were applied to synthesize different ocular staining scales, including SICCA ocular staining score, Oxford score, and van Bijsterveld score, the interpretation of pooled effect sizes should be approached cautiously because standardized estimates are difficult to translate into absolute clinical changes.Although SMDs were applied to synthesize different ocular staining scales, including SICCA ocular staining score, Oxford score, and van Bijsterveld score, because eligible RCTs reported these outcomes as numerical values with means and standard deviations, treating ordinal scores as continuous variables may introduce additional uncertainty. Therefore, pooled estimates should be interpreted cautiously. Third, emerging therapies for dry eye disease, such as lifitegrast and acoltremon, were not included because no eligible RCTs specifically enrolling patients with Sjögren’s syndrome–related dry eye disease were identified, and their comparative efficacy and safety in this population remain uncertain. Fourth, local inconsistency was detected in part of the TBUT network, and several adverse event estimates showed wide confidence intervals, limiting confidence in some treatment rankings. Potential outcome reporting bias cannot be completely excluded, particularly because some included studies involved commercially available products or industry support. Finally, OSDI was reported in only a small number of RCTs, and findings regarding subjective symptoms should therefore be considered exploratoryFuture studies should include larger head-to-head RCTs with standardized diagnostic criteria, treatment protocols, outcome definitions, and longer follow-up. More complete AE reporting is needed, especially for corticosteroids and immunomodulatory agents, and real-world studies may help clarify long-term effectiveness, tolerability, feasibility, and patient-perceived benefit.

## 5 Conclusion

This network meta-analysis indicates that topical ocular therapies may provide outcome-specific benefits for Sjögren’s syndrome–related dry eye disease. Platelet-rich plasma and autologous serum eye drops demonstrated favorable effects on ocular surface staining outcomes and tear film stability, whereas cyclosporine A showed potential benefits for tear secretion outcomes. Although tacrolimus was associated with a higher risk of reported adverse events compared with control, this finding should be interpreted cautiously because safety evidence was limited and mainly reflected short-term follow-up. Overall, these findings should be considered exploratory rather than a definitive hierarchy of topical therapies, given the sparse treatment network, limited direct comparisons, local inconsistency in the TBUT network, and incomplete adverse-event reporting. Further well-designed head-to-head randomized trials are warranted to clarify the comparative effectiveness and long-term safety of topical therapies for Sjögren’s syndrome–related ocular disease.

## Data Availability

The original contributions presented in the study are included in the article/**Supplementary Material**. Further inquiries can be directed to the corresponding authors.
