# Supplementary material for: Comparative efficacy and safety of topical therapies for ocular involvement in Sjögren’s syndrome: a systematic review and network meta-analysis
Source: Front Immunol. 2026 Aug 26;17:1899484. doi: 10.3389/fimmu.2026.1899484 (PMC13553919; doi:10.3389/fimmu.2026.1899484)
Supplement: Supplementary file 1 [file SupplementaryFile1.docx]

**Supplementary Appendix**

**Comparative Efficacy and Safety of Topical Ocular Therapies for Sjögren’s Syndrome–Related Dry Eye Disease: A Systematic Review and Network Meta-Analysis**

**Table of contents**

*Appendix 1: PRISMA-NMA checklist.................................................................................................................1*

*Appendix 2: Search strategy..............................................................................................................................7*

*Appendix 3: Characteristics of included studies .............................................................................................11*

*Appendix 4: Risk of bias of randomized clinical trials...................................................................................16*

*Appendix 5: Evaluation of consistency and heterogeneity .............................................................................19*

*Appendix 6: SUCRA and cumulative ranking probability plots.......................................................................24*

*Appendix 7: League table of summary estimates derived from network meta-analysis..................................32*

*Appendix 8: CINeMA Assessment ...................................................................................................................36*

*Appendix 9: Funnel plots.................................................................................................................................46*

*Appendix 10: Forest plots................................................................................................................................50*

*Appendix 11:* *Sensitivity analysis...................................................................................................................52*

**Appendix 1: PRISMA NMA Checklist**

| **Section/Topic** | **Item #** | **Checklist Item** | **Reported on Page #** |
| --- | --- | --- | --- |
| **TITLE** |  |  |  |
| Title | 1 | Identify the report as a systematic review *incorporating a network meta-analysis (or related form of meta-analysis).* | ***1*** |
|  |  |  |  |
| **ABSTRACT** |  |  |  |
| Structured summary | 2 | Provide a structured summary including, as applicable:  **Background:** main objectives  **Methods:** data sources; study eligibility criteria, participants, and interventions; study appraisal; and *synthesis methods, such as network meta-analysis.*  **Results:** number of studies and participants identified; summary estimates with corresponding confidence/credible intervals; *treatment rankings may also be discussed. Authors may choose to summarize pairwise comparisons against a chosen treatment included in their analyses for brevity.*  **Discussion/Conclusions:** limitations; conclusions and implications of findings.  **Other:** primary source of funding; systematic review registration number with registry name. | 2 |
|  |  |  |  |
| **INTRODUCTION** |  |  |  |
| Rationale | 3 | Describe the rationale for the review in the context of what is already known*, including mention of why a network meta-analysis has been conducted.* | ***3*** |
| Objectives | 4 | Provide an explicit statement of questions being addressed, with reference to participants, interventions, comparisons, outcomes, and study design (PICOS). | 4 |
|  |  |  |  |
| **METHODS** |  |  |  |
| Protocol and registration | 5 | Indicate whether a review protocol exists and if and where it can be accessed (e.g., Web address); and, if available, provide registration information, including registration number. | 4 |
| Eligibility criteria | 6 | Specify study characteristics (e.g., PICOS, length of follow-up) and report characteristics (e.g., years considered, language, publication status) used as criteria for eligibility, giving rationale. *Clearly describe eligible treatments included in the treatment network, and note whether any have been clustered or merged into the same node (with justification).* | 4 |
| Information sources | 7 | Describe all information sources (e.g., databases with dates of coverage, contact with study authors to identify additional studies) in the search and date last searched. | 4 |
| Search | 8 | Present full electronic search strategy for at least one database, including any limits used, such that it could be repeated. | 4 |
| Study selection | 9 | State the process for selecting studies (i.e., screening, eligibility, included in systematic review, and, if applicable, included in the meta-analysis). | 4 |
| Data collection process | 10 | Describe method of data extraction from reports (e.g., piloted forms, independently, in duplicate) and any processes for obtaining and confirming data from investigators. | 5 |
| Data items | 11 | List and define all variables for which data were sought (e.g., PICOS, funding sources) and any assumptions and simplifications made. | 5 |
| **Geometry of the network** | **S1** | Describe methods used to explore the geometry of the treatment network under study and potential biases related to it. This should include how the evidence base has been graphically summarized for presentation, and what characteristics were compiled and used to describe the evidence base to readers. | ***7*** |
| Risk of bias within individual studies | 12 | Describe methods used for assessing risk of bias of individual studies (including specification of whether this was done at the study or outcome level), and how this information is to be used in any data synthesis. | 6 |
| Summary measures | 13 | State the principal summary measures (e.g., risk ratio, difference in means). *Also describe the use of additional summary measures assessed, such as treatment rankings and surface under the cumulative ranking curve (SUCRA) values, as well as modified approaches used to present summary findings from meta-analyses.* | 7 |
| Planned methods of analysis | 14 | Describe the methods of handling data and combining results of studies for each network meta-analysis. This should include, but not be limited to:   - *Handling of multi-arm trials;* - *Selection of variance structure;* - *Selection of prior distributions in Bayesian analyses; and* - *Assessment of model fit.* | 7 |
| **Assessment of Inconsistency** | **S2** | Describe the statistical methods used to evaluate the agreement of direct and indirect evidence in the treatment network(s) studied. Describe efforts taken to address its presence when found. | 7 |
| Risk of bias across studies | 15 | Specify any assessment of risk of bias that may affect the cumulative evidence (e.g., publication bias, selective reporting within studies). | **6** |
| Additional analyses | 16 | Describe methods of additional analyses if done, indicating which were pre-specified. This may include, but not be limited to, the following:   - Sensitivity or subgroup analyses; - Meta-regression analyses; - *Alternative formulations of the treatment network; and* - *Use of alternative prior distributions for Bayesian analyses (if applicable).* | ***7*** |
| **RESULTS†** |  |  |  |
| Study selection | 17 | Give numbers of studies screened, assessed for eligibility, and included in the review, with reasons for exclusions at each stage, ideally with a flow diagram. | 8 |
| **Presentation of network structure** | **S3** | Provide a network graph of the included studies to enable visualization of the geometry of the treatment network. | ***Appendix 10*** |
| **Summary of network geometry** | **S4** | Provide a brief overview of characteristics of the treatment network. This may include commentary on the abundance of trials and randomized patients for the different interventions and pairwise comparisons in the network, gaps of evidence in the treatment network, and potential biases 7reflected by the network structure. | ***Appendix 10*** |
| Study characteristics | 18 | For each study, present characteristics for which data were extracted (e.g., study size, PICOS, follow-up period) and provide the citations. | 8-9 |
| Risk of bias within studies | 19 | Present data on risk of bias of each study and, if available, any outcome level assessment. | 9 |
| Results of individual studies | 20 | For all outcomes considered (benefits or harms), present, for each study: 1) simple summary data for each intervention group, and 2) effect estimates and confidence intervals. *Modified approaches may be needed to deal with information from larger networks.* | 9-12 |
| Synthesis of results | 21 | Present results of each meta-analysis done, including confidence/credible intervals. *In larger networks, authors may focus on comparisons versus a particular comparator (e.g. placebo or standard care), with full findings presented in an appendix. League tables and forest plots may be considered to summarize pairwise comparisons.* If additional summary measures were explored (such as treatment rankings), these should also be presented. | 9-12 |
| **Exploration for inconsistency** | **S5** | Describe results from investigations of inconsistency. This may include such information as measures of model fit to compare consistency and inconsistency models, *P* values from statistical tests, or summary of inconsistency estimates from different parts of the treatment network. | ***Appendix 5*** |
| Risk of bias across studies | 22 | Present results of any assessment of risk of bias across studies for the evidence base being studied. | 9 |
| Results of additional analyses | 23 | Give results of additional analyses, if done (e.g., sensitivity or subgroup analyses, meta-regression analyses*, alternative network geometries studied, alternative choice of prior distributions for Bayesian analyses,* and so forth). | **Not applicable** |
| **DISCUSSION** |  |  |  |
| Summary of evidence | 24 | Summarize the main findings, including the strength of evidence for each main outcome; consider their relevance to key groups (e.g., healthcare providers, users, and policy-makers). | 12-14 |
| Limitations | 25 | Discuss limitations at study and outcome level (e.g., risk of bias), and at review level (e.g., incomplete retrieval of identified research, reporting bias). *Comment on the validity of the assumptions, such as transitivity and consistency. Comment on any concerns regarding network geometry (e.g., avoidance of certain comparisons).* | 15-16 |
| Conclusions | 26 | Provide a general interpretation of the results in the context of other evidence, and implications for future research. | 16 |
| **FUNDING** |  |  |  |
| Funding | 27 | Describe sources of funding for the systematic review and other support (e.g., supply of data); role of funders for the systematic review. This should also include information regarding whether funding has been received from manufacturers of treatments in the network and/or whether some of the authors are content experts with professional conflicts of interest that could affect use of treatments in the network. | ***17*** |

**Appendix 2: Search strategy**

Table S1. Search strategy of PubMed

| # | Searches |
| --- | --- |
| 1 | (("Sjogren's Syndrome"[MeSH Terms]) OR (Sjogren*[Title/Abstract]) OR (Sjögren*[Title/Abstract]) OR ("sicca syndrome"[Title/Abstract]) OR ("primary Sjogren syndrome"[Title/Abstract]) OR ("primary Sjögren syndrome"[Title/Abstract]) OR ("secondary Sjogren syndrome"[Title/Abstract]) OR ("secondary Sjögren syndrome"[Title/Abstract])) |
| 2 | (("Dry Eye Syndromes"[MeSH Terms]) OR ("dry eye"[Title/Abstract]) OR ("dry eye disease"[Title/Abstract]) OR ("dry eye syndrome"[Title/Abstract]) OR ("keratoconjunctivitis sicca"[Title/Abstract]) OR ("ocular dryness"[Title/Abstract]) OR ("tear dysfunction"[Title/Abstract])) |
| 3 | (("Ophthalmic Solutions"[MeSH Terms]) OR ("Eye Drops"[MeSH Terms]) OR ("Artificial Tears"[MeSH Terms]) OR ("eye drop*"[Title/Abstract]) OR ("ophthalmic solution*"[Title/Abstract]) OR ("topical ocular"[Title/Abstract]) OR ("topical ophthalmic"[Title/Abstract]) OR ("artificial tear*"[Title/Abstract]) OR ("lubricant eye drop*"[Title/Abstract]) OR ("ocular lubricant*"[Title/Abstract]) OR ("hyaluronic acid"[Title/Abstract]) OR ("sodium hyaluronate"[Title/Abstract]) OR (carboxymethylcellulose[Title/Abstract]) OR (cyclosporine[Title/Abstract]) OR ("cyclosporin A"[Title/Abstract]) OR ("cyclosporine A"[Title/Abstract]) OR (tacrolimus[Title/Abstract]) OR (corticosteroid*[Title/Abstract]) OR (glucocorticoid*[Title/Abstract]) OR (loteprednol[Title/Abstract]) OR (fluorometholone[Title/Abstract]) OR (dexamethasone[Title/Abstract]) OR (prednisolone[Title/Abstract]) OR ("autologous serum"[Title/Abstract]) OR ("serum eye drop*"[Title/Abstract]) OR ("platelet-rich plasma"[Title/Abstract]) OR ("platelet rich plasma"[Title/Abstract]) OR (PRP[Title/Abstract]) OR (diquafosol[Title/Abstract]) OR (rebamipide[Title/Abstract]) OR (lifitegrast[Title/Abstract])) |
| 4 | (("Randomized Controlled Trial"[Publication Type]) OR ("Controlled Clinical Trial"[Publication Type]) OR (randomized[Title/Abstract]) OR (randomised[Title/Abstract]) OR (randomly[Title/Abstract]) OR (placebo[Title/Abstract]) OR (trial[Title/Abstract]) OR ("double blind"[Title/Abstract]) OR ("single blind"[Title/Abstract]) OR (RCT[Title/Abstract])) |
| 5 | #1 AND #2 AND #3 AND #4 |
| 6 | #5 NOT (animals[MeSH Terms] NOT humans[MeSH Terms]) |

Table S2. Search strategy of Embase

| # | Searches |
| --- | --- |
| 1 | (('sjogren syndrome'/exp) OR (sjogren*:ti,ab,kw) OR (sjögren*:ti,ab,kw) OR ('sicca syndrome':ti,ab,kw) OR ('primary sjogren syndrome':ti,ab,kw) OR ('primary sjögren syndrome':ti,ab,kw) OR ('secondary sjogren syndrome':ti,ab,kw) OR ('secondary sjögren syndrome':ti,ab,kw)) |
| 2 | (('dry eye disease'/exp) OR ('keratoconjunctivitis sicca'/exp) OR ('dry eye':ti,ab,kw) OR ('dry eye disease':ti,ab,kw) OR ('dry eye syndrome':ti,ab,kw) OR ('keratoconjunctivitis sicca':ti,ab,kw) OR ('ocular dryness':ti,ab,kw) OR ('tear dysfunction':ti,ab,kw)) |
| 3 | (('eye drop'/exp) OR ('ophthalmic solution'/exp) OR ('artificial tear'/exp) OR ('eye drop*':ti,ab,kw) OR ('ophthalmic solution*':ti,ab,kw) OR ('topical ocular':ti,ab,kw) OR ('topical ophthalmic':ti,ab,kw) OR ('artificial tear*':ti,ab,kw) OR ('lubricant eye drop*':ti,ab,kw) OR ('ocular lubricant*':ti,ab,kw) OR ('hyaluronic acid':ti,ab,kw) OR ('sodium hyaluronate':ti,ab,kw) OR (carboxymethylcellulose:ti,ab,kw) OR ('cyclosporine'/exp) OR (cyclosporine:ti,ab,kw) OR ('cyclosporin A':ti,ab,kw) OR ('cyclosporine A':ti,ab,kw) OR ('tacrolimus'/exp) OR (tacrolimus:ti,ab,kw) OR (corticosteroid*:ti,ab,kw) OR (glucocorticoid*:ti,ab,kw) OR (loteprednol:ti,ab,kw) OR (fluorometholone:ti,ab,kw) OR (dexamethasone:ti,ab,kw) OR (prednisolone:ti,ab,kw) OR ('autologous serum':ti,ab,kw) OR ('serum eye drop*':ti,ab,kw) OR ('platelet-rich plasma':ti,ab,kw) OR ('platelet rich plasma':ti,ab,kw) OR (PRP:ti,ab,kw) OR (diquafosol:ti,ab,kw) OR (rebamipide:ti,ab,kw) OR (lifitegrast:ti,ab,kw)) |
| 4 | (('randomized controlled trial'/exp) OR ('controlled clinical trial'/exp) OR (random*:ti,ab,kw) OR (placebo*:ti,ab,kw) OR (trial:ti,ab,kw) OR ('double blind':ti,ab,kw) OR ('single blind':ti,ab,kw) OR (RCT:ti,ab,kw)) |
| 5 | #1 AND #2 AND #3 AND #4 |
| 6 | #5 NOT ('animal'/exp NOT 'human'/exp) |

**Table S3. Search strategy of Cochrane Library**

| # | Searches |
| --- | --- |
| 1 | MeSH descriptor: [Sjogren's Syndrome] explode all trees |
| 2 | (Sjogren*:ti,ab,kw OR Sjögren*:ti,ab,kw OR "sicca syndrome":ti,ab,kw OR "primary Sjogren syndrome":ti,ab,kw OR "primary Sjögren syndrome":ti,ab,kw OR "secondary Sjogren syndrome":ti,ab,kw OR "secondary Sjögren syndrome":ti,ab,kw) |
| 3 | MeSH descriptor: [Dry Eye Syndromes] explode all trees |
| 4 | ("dry eye":ti,ab,kw OR "dry eye disease":ti,ab,kw OR "dry eye syndrome":ti,ab,kw OR "keratoconjunctivitis sicca":ti,ab,kw OR "ocular dryness":ti,ab,kw OR "tear dysfunction":ti,ab,kw) |
| 5 | MeSH descriptor: [Ophthalmic Solutions] explode all trees |
| 6 | ("eye drop*":ti,ab,kw OR "ophthalmic solution*":ti,ab,kw OR "topical ocular":ti,ab,kw OR "topical ophthalmic":ti,ab,kw OR "artificial tear*":ti,ab,kw OR "lubricant eye drop*":ti,ab,kw OR "ocular lubricant*":ti,ab,kw OR "hyaluronic acid":ti,ab,kw OR "sodium hyaluronate":ti,ab,kw OR carboxymethylcellulose:ti,ab,kw OR cyclosporine:ti,ab,kw OR "cyclosporin A":ti,ab,kw OR "cyclosporine A":ti,ab,kw OR tacrolimus:ti,ab,kw OR corticosteroid*:ti,ab,kw OR glucocorticoid*:ti,ab,kw OR loteprednol:ti,ab,kw OR fluorometholone:ti,ab,kw OR dexamethasone:ti,ab,kw OR prednisolone:ti,ab,kw OR "autologous serum":ti,ab,kw OR "serum eye drop*":ti,ab,kw OR "platelet-rich plasma":ti,ab,kw OR "platelet rich plasma":ti,ab,kw OR PRP:ti,ab,kw OR diquafosol:ti,ab,kw OR rebamipide:ti,ab,kw OR lifitegrast:ti,ab,kw) |
| 7 | (randomized:ti,ab,kw OR randomised:ti,ab,kw OR random*:ti,ab,kw OR placebo:ti,ab,kw OR trial:ti,ab,kw OR "double blind":ti,ab,kw OR "single blind":ti,ab,kw OR RCT:ti,ab,kw) |
| 8 | (#1 OR #2) AND (#3 OR #4) AND (#5 OR #6) AND #7 |

Table S4. Search strategy of Web of Science

| # | Searches |
| --- | --- |
| 1 | TS=("Sjogren*" OR "Sjögren*" OR "sicca syndrome" OR "primary Sjogren syndrome" OR "primary Sjögren syndrome" OR "secondary Sjogren syndrome" OR "secondary Sjögren syndrome") |
| 2 | TS=("dry eye" OR "dry eye disease" OR "dry eye syndrome" OR "keratoconjunctivitis sicca" OR "ocular dryness" OR "tear dysfunction") |
| 3 | TS=("eye drop*" OR "ophthalmic solution*" OR "topical ocular" OR "topical ophthalmic" OR "artificial tear*" OR "lubricant eye drop*" OR "ocular lubricant*" OR "hyaluronic acid" OR "sodium hyaluronate" OR carboxymethylcellulose OR cyclosporine OR "cyclosporin A" OR "cyclosporine A" OR tacrolimus OR corticosteroid* OR glucocorticoid* OR loteprednol OR fluorometholone OR dexamethasone OR prednisolone OR "autologous serum" OR "serum eye drop*" OR "platelet-rich plasma" OR "platelet rich plasma" OR PRP OR diquafosol OR rebamipide OR lifitegrast) |
| 4 | TS=(random* OR placebo* OR trial OR "controlled trial" OR "clinical trial" OR "double blind" OR "single blind" OR RCT) |
| 5 | #1 AND #2 AND #3 AND #4 |

Table S5. Search strategy of CNKI

| # | Searches |
| --- | --- |
| 1 | 主题 = (干燥综合征 OR 舍格伦综合征 OR 干燥症 OR Sjogren OR Sjögren) |
| 2 | 主题 = (干眼 OR 干眼症 OR 干眼病 OR 角结膜干燥症 OR 眼干) |
| 3 | 主题 = (人工泪液 OR 玻璃酸钠 OR 透明质酸钠 OR 羧甲基纤维素 OR 环孢素 OR 环孢素 A OR 他克莫司 OR 糖皮质激素 OR 皮质类固醇 OR 氟米龙 OR 氯替泼诺 OR 地塞米松 OR 泼尼松龙 OR 自体血清 OR 血清滴眼液 OR 富血小板血浆 OR PRP OR 二夸磷索 OR 瑞巴派特 OR 利非司特) |
| 4 | 主题 = (随机 OR 随机对照 OR 对照试验 OR 临床试验 OR 安慰剂 OR 双盲 OR 单盲) |
| 5 | #1 AND #2 AND #3 AND #4 |

Table S6. Search strategy of Wanfang Data

| # | Searches |
| --- | --- |
| 1 | 主题: (干燥综合征 OR 舍格伦综合征 OR 干燥症 OR Sjogren OR Sjögren) |
| 2 | 主题: (干眼 OR 干眼症 OR 干眼病 OR 角结膜干燥症 OR 眼干) |
| 3 | 主题: (人工泪液 OR 玻璃酸钠 OR 透明质酸钠 OR 羧甲基纤维素 OR 环孢素 OR 环孢素 A OR 他克莫司 OR 糖皮质激素 OR 皮质类固醇 OR 氟米龙 OR 氯替泼诺 OR 地塞米松 OR 泼尼松龙 OR 自体血清 OR 血清滴眼液 OR 富血小板血浆 OR PRP OR 二夸磷索 OR 瑞巴派特 OR 利非司特) |
| 4 | 主题: (随机 OR 随机对照 OR 对照试验 OR 临床试验 OR 安慰剂 OR 双盲 OR 单盲) |
| 5 | #1 AND #2 AND #3 AND #4 |

Table S7. Search strategy of VIP Database

| # | Searches |
| --- | --- |
| 1 | M = (干燥综合征 OR 舍格伦综合征 OR 干燥症 OR Sjogren OR Sjögren) |
| 2 | M = (干眼 OR 干眼症 OR 干眼病 OR 角结膜干燥症 OR 眼干) |
| 3 | M = (人工泪液 OR 玻璃酸钠 OR 透明质酸钠 OR 羧甲基纤维素 OR 环孢素 OR 环孢素 A OR 他克莫司 OR 糖皮质激素 OR 皮质类固醇 OR 氟米龙 OR 氯替泼诺 OR 地塞米松 OR 泼尼松龙 OR 自体血清 OR 血清滴眼液 OR 富血小板血浆 OR PRP OR 二夸磷索 OR 瑞巴派特 OR 利非司特) |
| 4 | M = (随机 OR 随机对照 OR 对照试验 OR 临床试验 OR 安慰剂 OR 双盲 OR 单盲) |
| 5 | #1 AND #2 AND #3 AND #4 |

Table S8. Search strategy of CBM

| # | Searches |
| --- | --- |
| 1 | ("干燥综合征"[常用字段] OR "舍格伦综合征"[常用字段] OR "干燥症"[常用字段] OR "Sjogren"[常用字段] OR "Sjögren"[常用字段]) |
| 2 | ("干眼"[常用字段] OR "干眼症"[常用字段] OR "干眼病"[常用字段] OR "角结膜干燥症"[常用字段] OR "眼干"[常用字段]) |
| 3 | ("人工泪液"[常用字段] OR "玻璃酸钠"[常用字段] OR "透明质酸钠"[常用字段] OR "羧甲基纤维素"[常用字段] OR "环孢素"[常用字段] OR "环孢素 A"[常用字段] OR "他克莫司"[常用字段] OR "糖皮质激素"[常用字段] OR "皮质类固醇"[常用字段] OR "氟米龙"[常用字段] OR "氯替泼诺"[常用字段] OR "地塞米松"[常用字段] OR "泼尼松龙"[常用字段] OR "自体血清"[常用字段] OR "血清滴眼液"[常用字段] OR "富血小板血浆"[常用字段] OR "PRP"[常用字段] OR "二夸磷索"[常用字段] OR "瑞巴派特"[常用字段] OR "利非司特"[常用字段]) |
| 4 | ("随机"[常用字段] OR "随机对照"[常用字段] OR "对照试验"[常用字段] OR "临床试验"[常用字段] OR "安慰剂"[常用字段] OR "双盲"[常用字段] OR "单盲"[常用字段]) |
| 5 | #1 AND #2 AND #3 AND #4 |

**Appendix** **3:** **Characteristics** **of** **included** **studies**

**Table S3.1:** Baseline of characteristics of included studies

| Author | Year | Country | Study type | Intervention mode | | Number of cases | | Age, years | | Gender, male/female | | Outcome indicators |
| --- | --- | --- | --- | --- | --- | --- | --- | --- | --- | --- | --- | --- |
|  |  |  |  | **Experimental** | **Control** | **Experimental** | **Control** | **Experimental** | **Control** | **Experimental** | **Control** |  |
| Yin | 2025 | China | RCT | TAC | CsA | 28 | 28 | 58.49 ± 5.82 | 57.67 ± 5.16 | 4/24 | 5/23 | 2, 3, 4, 5 |
| Chen | 2021 | China | RCT | CsA | CTL | 32 | 28 | 45.3 ± 8.5 | 44.2 ± 7.4 | 7/25 | 8/20 | 2, 5 |
| Aragona | 2013 | Italy | RCT | ST | CTL | 20 | 20 | 58.8 ± 6.7 | 60.1 ± 5.1 | 1/19 | 1/19 | 2, 3, 5 |
| Tauber | 2023 | United States | RCT | LAC（22μM） | CTL | 57 | 60 | 60.8 ± 11.2 | 60.0 ± 10.9 | 3/65 | 3/65 | 2, 5 |
| Tauber | 2023 | United States | RCT | LAC（44μM） | CTL | 60 | 60 | 60.1 ± 10.7 | 60.0 ± 10.9 | 2/66 | 3/65 | 2, 5 |
| Bachtalia | 2025 | Greece/Cyprus | RCT | AS | CTL | 10 | 10 | 61.4 ± 15.22 | 61.4 ± 15.22 | 0/10 | 0/10 | 2, 3, 4, 5 |
| Gao | 2023 | China | RCT | CsA | CTL | 20 | 20 | 51.4 ± 12.4 | 58.4 ± 9.55 | 2/18 | 1/19 | 1, 2, 3, 4, 5 |
| Gao | 2023 | China | RCT | CsA | CTL | 20 | 20 | 56.4 ± 12.4 | 58.4 ± 9.55 | 1/19 | 1/19 | 1, 2, 3, 4, 5 |
| Kang | 2023 | Republic of Korea | RCT | PRP | AS | 14 | 16 | 54.07 ± 11.283 | 54.56 ± 11.939 | 0/14 | 0/16 | 1, 2, 3, 4, 5 |
| Moscovici | 2015 | Brazil | RCT | TAC | CTL | 14 | 10 | 50.5 ± 9.65 | 50.5 ± 9.65 | — | — | 2, 3, 4, 5 |
| Moawad | 2021 | Egypt | RCT | TAC | CTL | 30 | 30 | 49.4 ± 12.92 | 49.4 ± 12.92 | 3/27 | 3/27 | 1, 2, 3, 4 |
| Moawad | 2021 | Egypt | RCT | CsA | CTL | 30 | 30 | 44.9 ± 12.58 | 44.9 ± 12.58 | 3/27 | 3/27 | 1, 2, 3, 4 |
| Lin | 2015 | China | RCT | ST | CsA | 19 | 16 | 50.42 ± 10.85 | 49.94 ± 10.74 | 0/19 | 0/16 | 2, 5 |
| Yang | 2022 | China | RCT | ST | CTL | 26 | 26 | 53.23 ± 11.75 | 51.85 ± 12.33 | 5/21 | 4/22 | 1, 3, 4, 5 |
| Liu | 2024 | China | RCT | CsA+DQS | CsA | 30 | 30 | 42.03 ± 6.58 | 43.44 ± 6.54 | 7/23 | 6/24 | 1, 2, 3, 5 |
| Liu | 2024 | China | RCT | CsA+DQS | DQS | 30 | 30 | 42.03 ± 6.58 | 42.59 ± 7.06 | 7/23 | 7/23 | 1, 2, 3, 5 |

**Note 1:** The two intervention columns list the treatment arms compared within each included study. Abbreviations: TAC, topical tacrolimus; CsA, topical cyclosporine A; CTL, control treatment, including artificial tears, lubricant eye drops, vehicle, placebo, PVP, sodium hyaluronate, or background control as defined in the original trial; ST, topical corticosteroid; LAC, Lacripep eye drops, with 22 and 44 μM doses combined; AS, autologous serum eye drops; PRP, platelet-rich plasma eye drops; DQS, topical diquafosol sodium; CsA+DQS, combined topical cyclosporine A and diquafosol sodium treatment.

**Note 2:** Outcome indicators: 1, Ocular Surface Disease Index; 2, ocular staining score; 3, tear breakup time; 4, Schirmer test; 5, adverse events.

**Table S3.2**. Assessment of potential effect modifiers related to the transitivity assumption

| Study | SS diagnostic criteria | SS subtype | Baseline dry eye severity | Concomitant treatments | Treatment duration | Follow-up time | Outcome measurement scales |
| --- | --- | --- | --- | --- | --- | --- | --- |
| Aragona et al. | AECG criteria | NR | Moderate-to-severe DED | Polyvinylpyrrolidone eye drops | 30 days | 45 days | TBUT; ocular staining |
| Lin et al. | AECG criteria | Primary/secondary SS | Moderate-to-severe DED | Sodium hyaluronate | 8 weeks | 8 weeks | OSS; OSDI; TBUT; Schirmer test |
| Moscovici et al. | SS diagnosis (criteria NR) | NR | SS-related DED | Vehicle control | 90 days | 90 days | Schirmer test; TBUT; ocular staining |
| Chen et al. | Confirmed SS diagnosis | NR | Moderate-to-severe DED | Artificial tears | 12 weeks | 14 days after withdrawal | OSS; TBUT; Schirmer test; OSDI |
| Moawad et al. | SS diagnosis | NR | Severe DED | Placebo control | 6 months | 6 months | OSDI; OSS; TBUT; Schirmer test |
| Gao et al. | Primary SS | Primary SS | Moderate-to-severe DED | Artificial tears | 12 weeks | 12 weeks | OSS; TBUT; Schirmer test; OSDI |
| Tauber et al. | AECG criteria | Primary SS | Moderate-to-severe ocular surface disease | Vehicle control | 28 days | 56 days | Ocular staining; TBUT; Schirmer test |
| Kang et al. | Primary SS diagnosis | Primary SS | Moderate-to-severe DED | None reported | 12 weeks | 12 weeks | Ocular staining; TBUT; Schirmer test; OSDI |
| Yang et al. | SS-related DED | NR | Moderate-to-severe DED | Sodium hyaluronate | 12 weeks | 12 weeks | TBUT; Schirmer test; OSDI |
| Liu et al. | SS-related moderate-to-severe DED | NR | Moderate-to-severe DED | Sodium hyaluronate | 12 weeks | 12 weeks | OSDI; ocular staining; TBUT-related assessment |
| Bachtalia et al. | Biopsy-proven SS | Primary/secondary SS | Severe refractory DED | Artificial tears | 3 months | 3 months | TBUT; Schirmer test; ocular staining |
| Yin et al. | SS-related severe DED | NR | Severe DED | None reported | 8 weeks | 8 weeks | TBUT; Schirmer test; OSS |

Abbreviations: SS, Sjögren’s syndrome; DED, dry eye disease; NR, not reported; AECG, American-European Consensus Group; OSS, ocular staining score; TBUT, tear break-up time; OSDI, Ocular Surface Disease Index.

Outcome measurement scales were reported according to the outcomes included in the network meta-analysis. Different ocular staining methods were considered potential sources of heterogeneity in ocular surface staining assessment.

Appendix 4: Risk of bias of randomized clinical trials

**Figure S4:** Overall risk of bias presented as percentage of each risk of bias item across all included studies. Green = Low risk, Red = High risk, Yellow = Some concerns.

Table S4.1: Study level risk of bias assessment using Cochrane risk of bias tool 2.0 for assessing risk of bias of randomized clinical trials.

| Study | D1 | D2 | D3 | D4 | D5 | Overall |
| --- | --- | --- | --- | --- | --- | --- |
| Yin 2025 | Low | Some concerns | Low | Some concerns | Low | Some concerns |
| Chen 2021 | Low | Low | Low | Low | Low | Low |
| Aragona 2013 | Low | Low | Low | Low | Low | Low |
| Tauber 2023 | Low | Low | Low | Low | Some concerns | Some concerns |
| Bachtalia 2025 | Low | Low | Low | Low | Some concerns | Some concerns |
| Gao 2023 | Low | Low | Low | Low | Low | Some concerns |
| Kang 2023 | Low | Low | Some concerns | Low | Some concerns | Some concerns |
| Moscovici 2015 | Low | Some concerns | High | Some concerns | Low | High |
| Moawad 2021 | Low | Some concerns | Low | Some concerns | Low | Some concerns |
| Lin 2015 | Low | High | Some concerns | High | Low | High |
| Yang 2022 | Low | Some concerns | Low | Some concerns | Low | Some concerns |
| Liu 2024 | Low | Some concerns | Low | Some concerns | Low | Some concerns |

| Yin 2025 | Some concerns | Low | Low | Low | Some concerns |
| --- | --- | --- | --- | --- | --- |

**Table S4.2**. Outcome-specific risk of bias assessment of included randomized controlled trials using the Cochrane Risk of Bias 2 (RoB 2) tool.

| Study | OSS | TBUT | Schirmer test | OSDI | Adverse events |
| --- | --- | --- | --- | --- | --- |
| Aragona 2013 | Some concerns | Low | Low | Some concerns | Low |
| Chen 2021 | Some concerns | Low | Low | Some concerns | Low |
| Tauber 2023 | Low | Some concerns | Low | Some concerns | Low |
| Bachtalia 2025 | Some concerns | Low | Low | Some concerns | Low |
| Gao 2023 | Low | Some concerns | Low | Some concerns | Low |
| Kang 2023 | Some concerns | Low | Low | Some concerns | Low |
| Moscovici 2015 | Some concerns | High | Low | Some concerns | Low |
| Moawad 2021 | Low | Low | Some concerns | Low | Low |
| Lin 2015 | Some concerns | Some concerns | Low | Some concerns | Low |
| Yang 2024 | Some concerns | Some concerns | Low | Some concerns | Low |
| Liu 2024 | Some concerns | Low | Low | Some concerns | Some concerns |

**Appendix 5: Evaluation of inconsistency and heterogeneity**

**Table S5.1: Global consistency**

| Clinical outcome | Chi square | P value | τ² |
| --- | --- | --- | --- |
| OSS | 1.43 | 0.4881 | 0.3452 |
| TBUT | 6.42 | 0.113 | 0.3273 |
| ST | 0.06 | 0.8019 | 0 |
| AEs | 1.11 | 0.5733 | 0 |

**Abbreviations:** OSS, Ocular Staining Score; TBUT, Tear Break-Up Time; ST, Schirmer Test; AEs, Adverse Events.

Table S5.2: Side-splitting analysis for inconsistency between direct and indirect comparisons in the network meta-analysis

(A) Ocular Staining Score (OSS)

| Comparison | Direct |  | Indirect |  | Difference |  |  |  |
| --- | --- | --- | --- | --- | --- | --- | --- | --- |
|  | Coef. | Std. Err. | Coef. | Std. Err. | Coef. | Std. Err. | P value | tau |
| CTL vs CsA | -3.0809 | 0.4980 | -1.8403 | 0.7729 | -1.2406 | 0.9221 | 0.1780 | 0.4304 |
| CTL vs TAC | -2.8000 | 0.8852 | -4.3073 | 0.8314 | 1.5073 | 1.2145 | 0.2150 | 0.4969 |
| CTL vs ST | -1.9000 | 1.0707 | -2.3812 | 1.0849 | 0.4812 | 1.5243 | 0.7520 | 0.7489 |
| CTL vs AS | -6.8000 | 1.2131 | 0.0000 | 99.9779 | -6.8000 | 99.9852 | 0.9460 | 0.6036 |
| CsA vs TAC | -1.3600 | 0.6846 | 0.1475 | 1.0031 | -1.5075 | 1.2144 | 0.2140 | 0.4969 |
| CsA vs ST | 0.3900 | 0.9337 | 0.8714 | 1.2048 | -0.4815 | 1.5243 | 0.7520 | 0.7489 |
| AS vs PRP | -0.1700 | 0.7555 | 13.4300 | 200.0576 | -13.6000 | 200.0580 | 0.9460 | 0.6036 |

**Note:** CTL = control group, including artificial tears, sodium hyaluronate, vehicle, placebo, PVP, or lubricant/background control; CsA = topical cyclosporine A; TAC = topical tacrolimus; ST = topical corticosteroid; AS = autologous serum eye drops; PRP = platelet-rich plasma eye drops.

(B) Tear Break-Up Time (TBUT)

| Comparison | Direct |  | Indirect |  | Difference |  |  |  |
| --- | --- | --- | --- | --- | --- | --- | --- | --- |
|  | Coef. | Std. Err. | Coef. | Std. Err. | Coef. | Std. Err. | P value | tau |
| CTL vs CsA | 0.9166 | 0.3178 | -0.8678 | 0.6285 | 1.7844 | 0.7043 | 0.0110 | 0.3484 |
| CTL vs TAC | 0.3021 | 0.4346 | 2.0865 | 0.5542 | -1.7844 | 0.7043 | 0.0110 | 0.3484 |
| CTL vs AS | 2.6526 | 0.9494 | 0.0000 | 99.9843 | 2.6526 | 99.9888 | 0.9790 | 0.6964 |
| CsA vs TAC | 1.1700 | 0.4540 | -0.6145 | 0.5384 | 1.7844 | 0.7043 | 0.0110 | 0.3484 |
| CsA vs LAC | -1.2965 | 0.7494 | -2.4357 | 200.1957 | 1.1392 | 200.1967 | 0.9950 | 0.6964 |
| CsA vs DQS | 2.5129 | 0.7675 | 1.3737 | 200.2397 | 1.1392 | 200.2407 | 0.9950 | 0.6964 |
| AS vs PRP | -0.0272 | 0.7867 | -5.3323 | 200.1315 | 5.3051 | 200.1324 | 0.9790 | 0.6964 |

**Note:** CTL = control group, including artificial tears, sodium hyaluronate, vehicle, placebo, PVP, or lubricant/background control; CsA = topical cyclosporine A; TAC = topical tacrolimus; AS = autologous serum eye drops; LAC = Lacripep eye drops; DQS = topical diquafosol sodium; PRP = platelet-rich plasma eye drops.

C) Schirmer Test

| Comparison | Direct |  | Indirect |  | Difference |  |  |  |
| --- | --- | --- | --- | --- | --- | --- | --- | --- |
|  | Coef. | Std. Err. | Coef. | Std. Err. | Coef. | Std. Err. | P value | tau |
| CTL vs CsA | 5.3626 | 0.8172 | 4.7979 | 2.0857 | 0.5647 | 2.2400 | 0.8010 | 0.0000 |
| CTL vs TAC | 3.2000 | 2.0186 | 3.7622 | 0.9721 | -0.5622 | 2.2405 | 0.8020 | 0.0000 |
| CTL vs AS | 3.6000 | 0.5979 | 0.0000 | 100.0120 | 3.6000 | 100.0138 | 0.9710 | 0.0000 |
| CsA vs TAC | -1.5999 | 0.5264 | -2.1635 | 2.1777 | 0.5635 | 2.2404 | 0.8010 | 0.0000 |
| AS vs PRP | -0.0200 | 0.9792 | -7.2200 | 200.0682 | 7.2000 | 200.0658 | 0.9710 | 0.0000 |

**Note:** CTL = control group, including artificial tears, sodium hyaluronate, vehicle, placebo, PVP, or lubricant/background control; CsA = topical cyclosporine A; TAC = topical tacrolimus; ST = topical corticosteroid; AS = autologous serum eye drops; PRP = platelet-rich plasma eye drops.

(D) Adverse Events

| Comparison | Direct |  | Indirect |  | Difference |  |  |  |
| --- | --- | --- | --- | --- | --- | --- | --- | --- |
|  | Coef. | Std. Err. | Coef. | Std. Err. | Coef. | Std. Err. | P value | tau |
| CTL vs CsA | 0.5350 | 0.9992 | 1.6140 | 0.9472 | -1.0791 | 1.3768 | 0.4330 | <0.0001 |
| CTL vs TAC | 1.7047 | 0.9170 | 1.4115 | 1.0495 | 0.2933 | 1.3937 | 0.8330 | <0.0001 |
| CTL vs ST | 0.0000 | 1.4261 | -2.0446 | 1.6882 | 2.0446 | 2.2100 | 0.3550 | <0.0001 |
| CTL vs AS | 0.0000 | 2.0471 | 0.0000 | 100.0088 | 0.0000 | 100.0298 | 1.0000 | <0.0001 |
| CsA vs TAC | 0.4308 | 0.5380 | 0.7240 | 1.2857 | -0.2932 | 1.3937 | 0.8330 | <0.0001 |
| CsA vs ST | -2.9257 | 1.5231 | -0.8816 | 1.6012 | -2.0441 | 2.2099 | 0.3550 | <0.0001 |
| CsA vs CsA+DQS | 0.0000 | 2.0163 | -2.2067 | 200.0070 | 2.2067 | 199.9968 | 0.9910 | <0.0001 |
| AS vs PRP | 0.1144 | 2.0285 | 0.1144 | 200.0413 | 0.0000 | 200.0311 | 1.0000 | <0.0001 |

**Note:** CTL = control group, including artificial tears, sodium hyaluronate, vehicle, placebo, PVP, or lubricant/background control; CsA = topical cyclosporine A; TAC = topical tacrolimus; ST = topical corticosteroid; AS = autologous serum eye drops; CsA+DQS = topical cyclosporine A plus diquafosol sodium; PRP = platelet-rich plasma eye drops

**Appendix 6: SUCRA and cumulative ranking probability plots**

**Figure S6.1:** Cumulative ranking probability plots of interventions for Ocular Staining Score (OSS) in the network meta-analysis. Because lower OSS values indicate better treatment effects, interventions were ranked in the direction of lower values. A higher SUCRA value therefore indicates a greater probability of being among the best-ranked interventions for reducing OSS.

**Note:** A = CTL; B = CsA; C = TAC; D = ST; E = AS; F = PRP. CTL = control group; CsA = topical cyclosporine A; TAC = topical tacrolimus; ST = topical corticosteroid; AS = autologous serum eye drops; PRP = platelet-rich plasma eye drops.

**Table S6.1: SUCRA of the effects of various interventions on Ocular Staining Score (OSS).**

| Treatment | SUCRA | PrBest | MeanRank |
| --- | --- | --- | --- |
| CTL | 0.0 | 0.0 | 6.0 |
| CsA | 37.9 | 0.0 | 4.1 |
| TAC | 57.6 | 0.5 | 3.1 |
| ST | 25.0 | 0.0 | 4.8 |
| AS | 87.9 | 40.3 | 1.6 |
| PRP | 91.6 | 59.1 | 1.4 |

**Abbreviations:** CTL, control group, including artificial tears, sodium hyaluronate, vehicle, placebo, PVP, or lubricant/background control; CsA, topical cyclosporine A; TAC, topical tacrolimus; ST, topical corticosteroid; AS, autologous serum eye drops; PRP, platelet-rich plasma eye drops; SUCRA, surface under the cumulative ranking curve; PrBest, probability of being ranked best.

**Figure S6.2:** Cumulative ranking probability plots of interventions for **Tear Break-Up Time (TBUT)** in the network meta-analysis. Because higher TBUT values indicate better treatment effects, interventions were ranked in the direction of higher values. A higher SUCRA value therefore indicates a greater probability of being among the best-ranked interventions for improving TBUT.

**Note:** A = CTL; B = CsA; C = TAC; D = ST; E = AS; F = PRP; H = DQS; I = CsA+DQS. CTL = control group, including artificial tears, sodium hyaluronate, vehicle, placebo, PVP, or lubricant/background control; CsA = topical cyclosporine A; TAC = topical tacrolimus; ST = topical corticosteroid; AS = autologous

serum eye drops; PRP = platelet-rich plasma eye drops; DQS = topical diquafosol sodium; CsA+DQS = topical cyclosporine A plus diquafosol sodium.

Table S6.2: SUCRA of the effects of various interventions on Tear Break-Up Time (TBUT).

| Treatment | SUCRA | PrBest | MeanRank |
| --- | --- | --- | --- |
| CTL | 4.4 | 0.0 | 7.7 |
| CsA | 38.8 | 0.0 | 5.3 |
| TAC | 52.1 | 0.4 | 4.4 |
| ST | 36.1 | 0.0 | 5.5 |
| AS | 90.5 | 44.5 | 1.7 |
| PRP | 87.2 | 42.7 | 1.9 |
| DQS | 15.3 | 0.0 | 6.9 |
| CsA+DQS | 75.6 | 12.4 | 2.7 |

**Abbreviations:** CTL, control group, including artificial tears, sodium hyaluronate, vehicle, placebo, PVP, or lubricant/background control; CsA, topical cyclosporine A; TAC, topical tacrolimus; ST, topical corticosteroid; AS, autologous serum eye drops; PRP, platelet-rich plasma eye drops; DQS, topical diquafosol sodium; CsA+DQS, topical cyclosporine A plus diquafosol sodium; SUCRA, surface under the cumulative ranking curve; PrBest, probability of being ranked best.

**Figure S6.3:** Cumulative ranking probability plots of interventions for **Schirmer Test** in the network meta-analysis. Because higher Schirmer Test values indicate better treatment effects, interventions were ranked in the direction of higher values. A higher SUCRA value therefore indicates a greater probability of being among the best-ranked interventions for improving tear secretion.

**Note:** A = CTL; B = CsA; C = TAC; D = ST; E = AS; F = PRP. CTL = control group, including artificial tears, sodium hyaluronate, vehicle, placebo, PVP, or lubricant/background control; CsA = topical cyclosporine A; TAC = topical tacrolimus; ST = topical corticosteroid; AS = autologous serum eye drops; PRP = platelet-rich plasma eye drops.

**Table S6.3: SUCRA of the effects of various interventions on Schirmer Test.**

| Treatment | SUCRA | PrBest | MeanRank |
| --- | --- | --- | --- |
| CTL | 0.9 | 0.0 | 6.0 |
| CsA | 97.1 | 88.0 | 1.1 |
| TAC | 60.9 | 0.1 | 3.0 |
| ST | 19.3 | 0.0 | 5.0 |
| AS | 60.3 | 1.7 | 3.0 |
| PRP | 61.5 | 10.2 | 2.9 |

**Abbreviations:** CTL, control group, including artificial tears, sodium hyaluronate, vehicle, placebo, PVP, or lubricant/background control; CsA, topical cyclosporine A; TAC, topical tacrolimus; ST, topical corticosteroid; AS, autologous serum eye drops; PRP, platelet-rich plasma eye drops; SUCRA, surface under the cumulative ranking curve; PrBest, probability of being ranked best.

**Figure S6.4:** Cumulative ranking probability plots of interventions for **Adverse Events (AEs)** in the network meta-analysis. Because lower rates of adverse events indicate better safety, interventions were ranked in the direction of lower values. A higher SUCRA value therefore indicates a greater probability of being among the best-ranked interventions with fewer adverse events.

**Note:** A = CTL; B = CsA; C = TAC; D = ST; E = AS; F = PRP; G = LAC; I = CsA+DQS. CTL = control group, including artificial tears, sodium hyaluronate, vehicle, placebo, PVP, or lubricant/background control; CsA = topical cyclosporine A; TAC = topical tacrolimus; ST = topical corticosteroid; AS = autologous serum eye drops; PRP = platelet-rich plasma eye drops; LAC = Lacripep eye drops; CsA+DQS = topical cyclosporine A plus diquafosol sodium.

**Table S6.4: SUCRA of the effects of various interventions on Adverse Events (AEs).**

| Treatment | SUCRA | PrBest | MeanRank |
| --- | --- | --- | --- |
| CTL | 62.6 | 3.1 | 3.6 |
| CsA | 36.8 | 0.4 | 5.4 |
| TAC | 25.4 | 0.1 | 6.2 |
| ST | 80.4 | 39.4 | 2.4 |
| AS | 52.7 | 13.1 | 4.3 |
| PRP | 50.7 | 25.3 | 4.5 |
| LAC | 47.6 | 2.5 | 4.7 |
| CsA+DQS | 43.8 | 16.2 | 4.9 |

**Abbreviations:** CTL, control group, including artificial tears, sodium hyaluronate, vehicle, placebo, PVP, or lubricant/background control; CsA, topical cyclosporine A; TAC, topical tacrolimus; ST, topical corticosteroid; AS, autologous serum eye drops; PRP, platelet-rich plasma eye drops; LAC, Lacripep eye drops; CsA+DQS, topical cyclosporine A plus diquafosol sodium; SUCRA, surface under the cumulative ranking curve; PrBest, probability of being ranked best.

Appendix 7: League table of summary estimates derived from network meta-analysis

**Table S7.1: Ocular Staining Score (OSS)**

Each cell represents the comparison between the intervention in the row and the intervention in the column. The effect estimates are expressed as mean differences (MDs) with 95% confidence intervals (CIs). For example, the MD in Ocular Staining Score for PRP compared with CTL is 6.96 (4.16, 9.76). Because lower OSS values indicate better treatment effects, positive MD values favor the intervention in the row, whereas negative MD values favor the intervention in the column. Green cells indicate statistically significant differences, where the 95% CI does not include 0.

| **CTL** |  |  |  |  |  |
| --- | --- | --- | --- | --- | --- |
| **2.72 (1.81, 3.63)** | **CsA** |  |  |  |  |
| **3.57 (2.26, 4.88)** | 0.86 (-0.38, 2.09) | **TAC** |  |  |  |
| **2.15 (0.81, 3.49)** | -0.57 (-1.86, 0.72) | -1.42 (-3.13, 0.28) | **ST** |  |  |
| **6.79 (4.42, 9.17)** | **4.08 (1.53, 6.62)** | **3.22 (0.51, 5.93)** | **4.64 (1.91, 7.37)** | **AS** |  |
| **6.96 (4.16, 9.76)** | **4.24 (1.30, 7.19)** | **3.39 (0.30, 6.48)** | **4.81 (1.71, 7.92)** | 0.17 (-1.31, 1.65) | **PRP** |

**Abbreviations:** CTL, control group; CsA, topical cyclosporine A; TAC, topical tacrolimus; ST, topical corticosteroid; AS, autologous serum eye drops; PRP, platelet-rich plasma eye drops; MD, mean difference; CI, confidence interval.

]

**Table S7.2: Tear Break-Up Time (TBUT)**

Each cell represents the comparison between the intervention in the row and the intervention in the column. The effect estimates are expressed as mean differences (MDs) with 95% confidence intervals (CIs). Because higher TBUT values indicate better treatment effects, **MD > 0 favors the intervention in the row**, whereas **MD < 0 favors the intervention in the column**. Green cells indicate statistically significant differences, where the 95% CI does not include 0.

| **CTL** |  |  |  |  |  |  |  |
| --- | --- | --- | --- | --- | --- | --- | --- |
| **-1.23 (-2.16, -0.30)** | **CsA** |  |  |  |  |  |  |
| **-1.68 (-2.86, -0.51)** | -0.45 (-1.47, 0.57) | **TAC** |  |  |  |  |  |
| **-1.13 (-2.08, -0.17)** | 0.10 (-1.23, 1.44) | 0.55 (-0.96, 2.07) | **ST** |  |  |  |  |
| **-3.90 (-5.57, -2.23)** | **-2.67 (-4.58, -0.76)** | **-2.22 (-4.26, -0.18)** | **-2.77 (-4.69, -0.85)** | **AS** |  |  |  |
| **-3.85 (-6.23, -1.47)** | **-2.62 (-5.18, -0.06)** | -2.17 (-4.82, 0.49) | **-2.72 (-5.29, -0.16)** | 0.05 (-1.65, 1.75) | **PRP** |  |  |
| -0.42 (-1.90, 1.06) | 0.81 (-0.35, 1.97) | 1.26 (-0.28, 2.81) | 0.71 (-1.06, 2.47) | **3.48 (1.25, 5.71)** | **3.43 (0.62, 6.24)** | **DQS** |  |
| **-2.80 (-4.29, -1.31)** | **-1.57 (-2.74, -0.40)** | -1.12 (-2.67, 0.43) | -1.67 (-3.44, 0.10) | 1.10 (-1.14, 3.34) | 1.05 (-1.76, 3.86) | **-2.38 (-4.00, -0.76)** | **CsA+DQS** |

**Abbreviations:** CTL, control group, including artificial tears, sodium hyaluronate, vehicle, placebo, PVP, or lubricant/background control; CsA, topical cyclosporine A; TAC, topical tacrolimus; ST, topical corticosteroid; AS, autologous serum eye drops; PRP, platelet-rich plasma eye drops; DQS, topical diquafosol sodium; CsA+DQS, topical cyclosporine A plus diquafosol sodium; TBUT, tear break-up time; MD, mean difference; CI, confidence interval.

**Table S7.3: Schirmer I Test (SIT)**
Each cell represents the comparison between the intervention in the row and the intervention in the column. The effect estimates are expressed as mean differences (MDs) with 95% confidence intervals (CIs). Because higher Schirmer I Test values indicate better tear production and improved treatment effects, MD > 0 favors the intervention in the row, whereas MD < 0 favors the intervention in the column. Green cells indicate statistically significant differences, where the 95% CI does not include 0.

| **CTL** |  |  |  |  |  |
| --- | --- | --- | --- | --- | --- |
| **-5.29 (-6.78, -3.80)** | **CsA** |  |  |  |  |
| **-3.66 (-5.37, -1.94)** | **1.63 (0.63, 2.63)** | **TAC** |  |  |  |
| -0.60 (-1.28, 0.08) | **4.69 (3.05, 6.33)** | **3.06 (1.21, 4.90)** | **ST** |  |  |
| **-3.60 (-4.77, -2.43)** | 1.69 (-0.21, 3.58) | 0.06 (-2.02, 2.13) | **-3.00 (-4.36, -1.64)** | **AS** |  |
| **-3.58 (-5.83, -1.33)** | 1.71 (-0.99, 4.41) | 0.08 (-2.75, 2.91) | **-2.98 (-5.33, -0.63)** | 0.02 (-1.90, 1.94) | **PRP** |

Abbreviations: CTL, control group, including artificial tears, sodium hyaluronate, vehicle, placebo, PVP, or lubricant/background control; CsA, topical cyclosporine A; TAC, topical tacrolimus; ST, topical corticosteroid; AS, autologous serum eye drops; PRP, platelet-rich plasma eye drops;

**Table S7.4: Adverse Events (AEs)**

The lower triangle presents pairwise comparisons between interventions. Effect estimates are expressed as odds ratios (ORs) with 95% confidence intervals (CIs). Because lower adverse-event rates indicate better safety, an OR < 1 favors the intervention in the row, whereas an OR > 1 favors the intervention in the column. Green cells indicate statistically significant differences, where the 95% CI does not include 1.

| **CTL** |  |  |  |  |  |  |  |
| --- | --- | --- | --- | --- | --- | --- | --- |
| 0.57 (0.24, 1.33) | **CsA** |  |  |  |  |  |  |
| **0.47 (0.23, 0.99)** | 0.83 (0.49, 1.40) | **TAC** |  |  |  |  |  |
| 2.65 (0.35, 19.78) | 4.64 (0.62, 34.85) | 5.60 (0.74, 42.55) | **ST** |  |  |  |  |
| 1.00 (0.02, 46.04) | 1.75 (0.03, 88.56) | 2.12 (0.04, 104.54) | 0.38 (0.00, 28.58) | **AS** |  |  |  |
| 0.89(0.00, 206.34) | 1.57 (0.01, 386.28) | 1.89 (0.01, 458.97) | 0.34 (0.00, 111.76) | 0.89 (0.02, 42.68) | **PRP** |  |  |
| 0.77 (0.29, 2.07) | 1.35 (0.37, 4.96) | 1.63 (0.47, 5.59) | 0.29 (0.03, 2.74) | 0.77 (0.01, 40.16) | 0.86 (0.00, 216.77) | **LAC** |  |
| 0.57 (0.01, 30.50) | 1.00 (0.02, 48.82) | 1.21 (0.02, 61.08) | 0.22 (0.00, 17.20) | 0.57 (0.00, 142.74) | 0.64 (0.00, 539.26) | 0.74 (0.01, 44.75) | **CsA+DQS** |

Abbreviations: CTL, control group, including artificial tears, sodium hyaluronate, vehicle, placebo, PVP, or lubricant/background control; CsA, topical cyclosporine A; TAC, topical tacrolimus; ST, topical corticosteroid; AS, autologous serum eye drops; PRP, platelet-rich plasma eye drops; LAC, Lacripep eye drops; CsA+DQS, topical cyclosporine A plus diquafosol sodium; AEs, adverse events; RR, risk ratio; CI, confidence interval.

**Appendix 8: CINeMA Assessment**

We used the CINeMA framework to assess the certainty of evidence for each network estimate across four clinical outcomes: Ocular Staining, TBUT, Schirmer test, and Adverse Events. The assessment was based on the following domains:

**A. Within-study bias:**
The overall risk of bias for each included study was classified as low, moderate, or high according to the four assessed risk-of-bias items. Studies were considered to have a low overall risk of bias when all four items were rated as low risk, a moderate risk of bias when at least one item was rated as moderate risk but none was rated as high risk, and a high risk of bias when at least one item was rated as high risk. Details of the risk-of-bias assessment are provided in Appendix 8. The risk-of-bias contribution for each network comparison and the overall risk of bias by treatment comparison are shown in Figures S8.1–S8.8.

**Figure S8.1:** Risk of bias contribution by intervention group in Ocular Staining

**Figure S8.2:** Overall risk of bias by treatment comparison in Ocular Staining

**Figure S8.3:** Risk of bias contribution by intervention group in TBUT

**Figure S8.4:** Overall risk of bias by treatment comparison in TBUT

**Figure S8.5:** Risk of bias contribution by intervention group in Schirmer test

**Figure S8.6:** Overall risk of bias by treatment comparison in Schirmer test

**Figure S8.7:** Risk of bias contribution by intervention group in Adverse Events

**Figure S8.8:** Overall risk of bias by treatment comparison in Adverse Events

**Appendix 8: CINeMA Assessment**

We used the CINeMA framework to assess the certainty of evidence for each network estimate across four clinical outcomes: Ocular Staining, TBUT, Schirmer test, and Adverse Events. The assessment was based on within-study bias, reporting bias, imprecision, heterogeneity, and incoherence.

**The CINeMA assessment results for each clinical outcome are shown in Tables S8.1–S8.4.**

| Comparison | Within-study bias | Reporting bias | Indirectness | Imprecision | Heterogeneity | Incoherence | Confidence rating |
| --- | --- | --- | --- | --- | --- | --- | --- |
| AS:CTL | Some concerns | Low risk | No concerns | No concerns | No concerns | No concerns | Moderate |
| AS:PRP | Some concerns | Low risk | No concerns | Major concerns | No concerns | No concerns | Low |
| CTL:CsA | Some concerns | Low risk | No concerns | No concerns | No concerns | No concerns | Moderate |
| CTL:ST | No concerns | Low risk | No concerns | No concerns | Some concerns | No concerns | Moderate |
| CTL:TAC | Some concerns | Low risk | No concerns | No concerns | No concerns | No concerns | Moderate |
| CsA:ST | Major concerns | Low risk | No concerns | Some concerns | Some concerns | No concerns | Low |
| CsA:TAC | Some concerns | Low risk | No concerns | Some concerns | Some concerns | No concerns | Moderate |
| AS:CsA | Some concerns | Low risk | No concerns | No concerns | No concerns | No concerns | Moderate |
| AS:ST | Some concerns | Low risk | No concerns | No concerns | No concerns | No concerns | Moderate |
| AS:TAC | Some concerns | Low risk | No concerns | No concerns | Some concerns | No concerns | Moderate |
| CTL:PRP | Some concerns | Low risk | No concerns | No concerns | No concerns | No concerns | Moderate |
| CsA:PRP | Some concerns | Low risk | No concerns | No concerns | Some concerns | No concerns | Moderate |
| PRP:ST | Some concerns | Low risk | No concerns | No concerns | No concerns | No concerns | Moderate |
| PRP:TAC | Some concerns | Low risk | No concerns | No concerns | Major concerns | No concerns | Low |
| ST:TAC | Some concerns | Low risk | No concerns | Some concerns | Some concerns | No concerns | Moderate |

Table S8.1. CINeMA results for Ocular Staining

**Note:** AS = autologous serum; CTL = control; PRP = platelet-rich plasma; CsA = cyclosporine A; ST = steroid treatment; TAC = tacrolimus; TBUT = tear break-up time; AEs = adverse events; CINeMA = Confidence in Network Meta-Analysis; CI = confidence interval.

**Table S8.2. CINeMA results for TBUT**

| Comparison | Within-study bias | Reporting bias | Indirectness | Imprecision | Heterogeneity | Incoherence | Confidence rating |
| --- | --- | --- | --- | --- | --- | --- | --- |
| AS:CTL | Some concerns | Low risk | No concerns | No concerns | Some concerns | Major concerns | Moderate |
| AS:PRP | Some concerns | Low risk | No concerns | Major concerns | No concerns | Major concerns | Low |
| CTL:CsA | Some concerns | Low risk | No concerns | No concerns | Major concerns | No concerns | Moderate |
| CTL:ST | No concerns | Low risk | No concerns | Some concerns | Some concerns | Major concerns | Low |
| CTL:TAC | Some concerns | Low risk | No concerns | No concerns | Major concerns | No concerns | Moderate |
| CsA:CsA+DQS | Some concerns | Low risk | No concerns | Some concerns | Some concerns | Major concerns | Low |
| CsA:DQS | Some concerns | Low risk | No concerns | Major concerns | No concerns | Major concerns | Low |
| CsA:TAC | Some concerns | Low risk | No concerns | Major concerns | No concerns | No concerns | Low |
| CsA+DQS:DQS | Some concerns | Low risk | No concerns | No concerns | Major concerns | Major concerns | Moderate |
| AS:CsA | Some concerns | Low risk | No concerns | Some concerns | Some concerns | Major concerns | Moderate |
| AS:CsA+DQS | Some concerns | Low risk | No concerns | Major concerns | No concerns | Major concerns | Low |
| AS:DQS | Some concerns | Low risk | No concerns | Some concerns | Some concerns | Major concerns | Low |
| AS:ST | Some concerns | Low risk | No concerns | No concerns | Major concerns | Major concerns | Moderate |
| AS:TAC | Some concerns | Low risk | No concerns | Some concerns | Some concerns | Major concerns | Low |
| CTL:CsA+DQS | Some concerns | Low risk | No concerns | No concerns | Major concerns | Major concerns | Moderate |
| CTL:DQS | Some concerns | Low risk | No concerns | Major concerns | No concerns | Major concerns | Low |
| CTL:PRP | Some concerns | Low risk | No concerns | No concerns | Major concerns | Major concerns | Moderate |
| CsA:PRP | Some concerns | Low risk | No concerns | Major concerns | No concerns | Major concerns | Low |
| CsA:ST | Some concerns | Low risk | No concerns | Major concerns | No concerns | Major concerns | Low |
| CsA+DQS:PRP | Some concerns | Low risk | No concerns | Major concerns | No concerns | Major concerns | Low |
| CsA+DQS:ST | Some concerns | Low risk | No concerns | Some concerns | Some concerns | Major concerns | Low |
| CsA+DQS:TAC | Some concerns | Low risk | No concerns | Major concerns | No concerns | Major concerns | Low |
| DQS:PRP | Some concerns | Low risk | No concerns | Major concerns | No concerns | Major concerns | Low |
| DQS:ST | Some concerns | Low risk | No concerns | Major concerns | No concerns | Major concerns | Low |
| DQS:TAC | Some concerns | Low risk | No concerns | Major concerns | No concerns | Major concerns | Low |
| PRP:ST | Some concerns | Low risk | No concerns | Some concerns | Some concerns | Major concerns | Low |
| PRP:TAC | Some concerns | Low risk | No concerns | Major concerns | No concerns | Major concerns | Low |
| ST:TAC | Some concerns | Low risk | No concerns | Major concerns | No concerns | Major concerns | Low |

**Note:** AS = autologous serum; CTL = control; PRP = platelet-rich plasma; CsA = cyclosporine A; DQS = diquafosol sodium; CsA+DQS = cyclosporine A plus diquafosol sodium; ST = steroid treatment; TAC = tacrolimus; CINeMA = Confidence in Network Meta-Analysis. Treatment comparisons are presented as Intervention A:Intervention B. The confidence rating was graded as high, moderate, low, or very low according to the CINeMA assessment domains, including within-study bias, reporting bias, indirectness, imprecision, heterogeneity, and incoherence.

**Table S8.3. CINeMA results for Schirmer test**

| Comparison | Within-study bias | Reporting bias | Indirectness | Imprecision | Heterogeneity | Incoherence | Confidence rating |
| --- | --- | --- | --- | --- | --- | --- | --- |
| AS:CTL | Some concerns | Low risk | No concerns | No concerns | No concerns | No concerns | Moderate |
| AS:PRP | Some concerns | Low risk | No concerns | Major concerns | No concerns | No concerns | Low |
| CTL:CsA | Some concerns | Low risk | No concerns | No concerns | No concerns | No concerns | Moderate |
| CTL:ST | Some concerns | Low risk | No concerns | Some concerns | No concerns | No concerns | Moderate |
| CTL:TAC | Some concerns | Low risk | No concerns | No concerns | Some concerns | No concerns | Moderate |
| CsA:TAC | Some concerns | Low risk | No concerns | No concerns | Some concerns | No concerns | Moderate |
| AS:CsA | Some concerns | Low risk | No concerns | Some concerns | Some concerns | No concerns | Moderate |
| AS:ST | Some concerns | Low risk | No concerns | No concerns | No concerns | No concerns | Moderate |
| AS:TAC | Some concerns | Low risk | No concerns | Major concerns | No concerns | No concerns | Low |
| CTL:PRP | Some concerns | Low risk | No concerns | No concerns | Major concerns | No concerns | Low |
| CsA:PRP | Some concerns | Low risk | No concerns | Some concerns | Some concerns | No concerns | Moderate |
| CsA:ST | Some concerns | Low risk | No concerns | No concerns | No concerns | No concerns | Moderate |
| PRP:ST | Some concerns | Low risk | No concerns | No concerns | Major concerns | No concerns | Low |
| PRP:TAC | Some concerns | Low risk | No concerns | Major concerns | No concerns | No concerns | Low |
| ST:TAC | Some concerns | Low risk | No concerns | No concerns | Some concerns | No concerns | Moderate |

**Note:** AS = autologous serum; CTL = control; PRP = platelet-rich plasma; CsA = cyclosporine A; ST = steroid treatment; TAC = tacrolimus; CINeMA = Confidence in Network Meta-Analysis. Treatment comparisons are presented as Intervention A:Intervention B. The confidence rating was graded according to the CINeMA assessment domains, including within-study bias, reporting bias, imprecision, heterogeneity, and incoherence.

**Table S8.4. CINeMA results for Adverse Events**

| Comparison | Within-study bias | Reporting bias | Indirectness | Imprecision | Heterogeneity | Incoherence | Confidence rating |
| --- | --- | --- | --- | --- | --- | --- | --- |
| AS:CTL | Some concerns | Low risk | No concerns | Major concerns | No concerns | No concerns | Low |
| AS:PRP | Some concerns | Low risk | No concerns | Major concerns | No concerns | No concerns | Low |
| CTL:CsA | Some concerns | Low risk | No concerns | Major concerns | No concerns | No concerns | Low |
| CTL:LAC | Some concerns | Low risk | No concerns | Major concerns | No concerns | No concerns | Low |
| CTL:ST | Some concerns | Low risk | No concerns | Major concerns | No concerns | No concerns | Low |
| CTL:TAC | Major concerns | Low risk | No concerns | No concerns | Major concerns | No concerns | Moderate |
| CsA:CsA+DQS | Some concerns | Low risk | No concerns | Major concerns | No concerns | No concerns | Low |
| CsA:ST | Major concerns | Low risk | No concerns | Major concerns | No concerns | No concerns | Low |
| CsA:TAC | Some concerns | Low risk | No concerns | Major concerns | No concerns | No concerns | Low |
| AS:CsA | Some concerns | Low risk | No concerns | Major concerns | No concerns | No concerns | Low |
| AS:CsA+DQS | Some concerns | Low risk | No concerns | Major concerns | No concerns | No concerns | Low |
| AS:LAC | Some concerns | Low risk | No concerns | Major concerns | No concerns | No concerns | Low |
| AS:ST | Some concerns | Low risk | No concerns | Major concerns | No concerns | No concerns | Low |
| AS:TAC | Some concerns | Low risk | No concerns | Major concerns | No concerns | No concerns | Low |
| CTL:CsA+DQS | Some concerns | Low risk | No concerns | Major concerns | No concerns | No concerns | Low |
| CTL:PRP | Some concerns | Low risk | No concerns | Major concerns | No concerns | No concerns | Low |
| CsA:LAC | Some concerns | Low risk | No concerns | Major concerns | No concerns | No concerns | Low |
| CsA:PRP | Some concerns | Low risk | No concerns | Major concerns | No concerns | No concerns | Low |
| CsA+DQS:LAC | Some concerns | Low risk | No concerns | Major concerns | No concerns | No concerns | Low |
| CsA+DQS:PRP | Some concerns | Low risk | No concerns | Major concerns | No concerns | No concerns | Low |
| CsA+DQS:ST | Some concerns | Low risk | No concerns | Major concerns | No concerns | No concerns | Low |
| CsA+DQS:TAC | Some concerns | Low risk | No concerns | Major concerns | No concerns | No concerns | Low |
| LAC:PRP | Some concerns | Low risk | No concerns | Major concerns | No concerns | No concerns | Low |
| LAC:ST | Some concerns | Low risk | No concerns | Major concerns | No concerns | No concerns | Low |
| LAC:TAC | Some concerns | Low risk | No concerns | Major concerns | No concerns | No concerns | Low |
| PRP:ST | Some concerns | Low risk | No concerns | Major concerns | No concerns | No concerns | Low |
| PRP:TAC | Some concerns | Low risk | No concerns | Major concerns | No concerns | No concerns | Low |
| ST:TAC | Major concerns | Low risk | No concerns | Major concerns | No concerns | No concerns | Low |

**Note:** AS = autologous serum; CTL = control; PRP = platelet-rich plasma; CsA = cyclosporine A; DQS = diquafosol sodium; CsA+DQS = cyclosporine A plus diquafosol sodium; LAC = [please define according to the intervention used in this study]; ST = steroid treatment; TAC = tacrolimus; CINeMA = Confidence in Network Meta-Analysis. Treatment comparisons are presented as Intervention A:Intervention B. The confidence rating was graded according to the CINeMA assessment domains, including within-study bias, reporting bias, imprecision, heterogeneity, and incoherence.

**Appendix 9: Funnel plots**

The figures present an assessment of small-study effects and potential publication bias across studies investigating the efficacy and safety of different interventions for dry eye disease. Funnel plots were generated separately for the four clinical outcomes: **Ocular Staining, TBUT, Schirmer test, and Adverse Events**.

For studies with multi-arm designs, comparison results between each eligible intervention group and the corresponding control or comparator group were plotted separately. Egger’s linear regression test was used to quantitatively assess funnel plot asymmetry. A P value > 0.05 was interpreted as indicating no significant evidence of funnel plot asymmetry and a low likelihood of publication bias. When the P value was < 0.05, the trim-and-fill method was further applied to adjust the pooled effect estimate and assess the robustness of the findings.

**Figure S9.1. Funnel plot for Ocular Staining**

**Figure S9.2. Funnel plot for TBUT**

**Figure S9.3. Funnel plot for Schirmer test**

**Figure S9.4. Funnel plot for Adverse Events**

**Appendix 10: Forest plots**

Forest plots were generated to visually present the effect estimates of each intervention compared with the control group across four clinical outcomes: **Ocular Staining, TBUT, Schirmer test, and Adverse Events**. These plots summarize the relative treatment effects and corresponding 95% confidence intervals for each intervention-versus-control comparison.

For continuous outcomes, including Ocular Staining, TBUT, and Schirmer test, effect sizes were presented as mean differences or standardized mean differences, depending on the measurement scale used across studies. For dichotomous outcomes, including Adverse Events, effect sizes were presented as odds ratios or risk ratios. The control group was used as the common reference comparator in all forest plots.

The forest plots were used to assess the direction, magnitude, and precision of treatment effects. Estimates with confidence intervals crossing the null value were interpreted as not statistically significant, whereas estimates with confidence intervals not crossing the null value were considered statistically significant. Wider confidence intervals indicated greater uncertainty in the estimated treatment effect.

**Note:** TBUT = tear break-up time. For Ocular Staining, TBUT, and Schirmer test, treatment effects were reported as continuous effect estimates. For Adverse Events, treatment effects were reported as dichotomous effect estimates. The control group was used as the reference comparator in all analyses. The null value was 0 for mean difference or standardized mean difference and 1 for odds ratio or risk ratio.

**Figure S10.1. Forest plot of interventions versus control for Ocular Staining**

**Figure S10.2. Forest plot of interventions versus control for TBUT**

**Figure S10.3. Forest plot of interventions versus control for Schirmer test**

**Figure S10.4. Forest plot of interventions versus control for Adverse Events**

**Appendix 11. Sensitivity analysis using alternative control definitions**

**Note:** Sensitivity analyses were performed by restricting the control node (CTL) to inactive comparators (placebo or vehicle controls) to evaluate the potential impact of heterogeneous control definitions on network estimates. Treatment effects were compared with the primary analysis.

**Table S11.1. Sensitivity analysis of ocular staining score (OSS)**

| **Comparison** | **Main analysis SMD (95% CI)** | **Sensitivity analysis SMD (95% CI)** |
| --- | --- | --- |
| TAC vs CTL | -3.57 (-4.88, -2.26) | -2.95 (-3.87, -2.02) |
| CsA vs CTL | -2.72 (-3.63, -1.81) | -4.31 (-5.94, -2.68) |
| ST vs CTL | -2.15 (-3.49, -0.81) | -2.29 (-3.57, -1.02) |
| AS vs CTL | -6.79 (-9.17, -4.42) | -6.79 (-9.07, -4.51) |
| PRP vs CTL | -6.96 (-9.76, -4.16) | -6.96 (-9.60, -4.33) |

**Abbreviations:** OSS, ocular staining score; SMD, standardized mean difference; CI, confidence interval; CTL, control treatment; TAC, topical tacrolimus; CsA, topical cyclosporine A; ST, topical corticosteroid; AS, autologous serum; PRP, platelet-rich plasma.

**Table S11.2. Sensitivity analysis of tear break-up time (TBUT)**

| **Comparison** | **Main analysis MD (95% CI)** | **Sensitivity analysis MD (95% CI)** |
| --- | --- | --- |
| CsA vs CTL | 1.23 (0.30, 2.16) | 1.44 (0.11, 2.76) |
| TAC vs CTL | 1.68 (0.45, 2.91) | 2.61 (0.33, 4.88) |
| ST vs CTL | 1.13 (0.18, 2.08) | 0.66 (-0.64, 1.97) |
| AS vs CTL | 3.90 (2.24, 5.56) | 2.65 (0.48, 4.82) |
| PRP vs CTL | 3.85 (1.48, 6.22) | 2.62 (-0.26, 5.51) |
| DQS vs CTL | 0.42 (-1.05, 1.89) | 0.80 (-0.80, 2.40) |
| CsA+DQS vs CTL | 2.80 (1.32, 4.28) | 2.51 (0.64, 4.39) |

**Abbreviations:** TBUT, tear break-up time; MD, mean difference; CI, confidence interval; CTL, control treatment; CsA, cyclosporine A; TAC, tacrolimus; ST, topical corticosteroid; AS, autologous serum; PRP, platelet-rich plasma; DQS, diquafosol sodium; CsA+DQS, combined cyclosporine A and diquafosol sodium treatment.

**Table S11.3. Sensitivity analysis of Schirmer test**

| **Comparison** | **Main analysis MD (95% CI)** | **Sensitivity analysis MD (95% CI)** |
| --- | --- | --- |
| CsA vs CTL | 5.29 (3.80, 6.78) | 5.36 (3.76, 6.96) |
| TAC vs CTL | 3.66 (1.94, 5.37) | 3.76 (1.86, 5.67) |
| ST vs CTL | 0.60 (-0.08, 1.28) | 0.60 (-0.08, 1.28) |
| AS vs CTL | 3.60 (2.43, 4.77) | 3.60 (2.43, 4.77) |
| PRP vs CTL | 3.58 (1.33, 5.83) | 3.58 (1.33, 5.83) |

**Abbreviations:** MD, mean difference; CI, confidence interval; CTL, control treatment; CsA, cyclosporine A; TAC, tacrolimus; ST, topical corticosteroid; AS, autologous serum; PRP, platelet-rich plasma.

**Table S11.4. Sensitivity analysis of adverse events**

| **Comparison** | **Main analysis OR (95% CI)** | **Sensitivity analysis OR (95% CI)** |
| --- | --- | --- |
| CsA vs CTL | 1.75 (0.75, 4.09) | 3.01 (0.78, 11.59) |
| TAC vs CTL | 2.12 (1.01, 4.42) | 4.84 (1.25, 18.75) |
| ST vs CTL | 0.38 (0.05, 2.83) | 0.43 (0.05, 3.61) |
| AS vs CTL | 1.00 (0.02, 46.04) | 1.00 (0.02, 55.25) |
| PRP vs CTL | 1.12 (0.00, 257.74) | 1.12 (0.00, 318.11) |
| LAC vs CTL | 1.30 (0.48, 3.50) | 1.33 (0.45, 3.90) |
| CsA+DQS vs CTL | 1.75 (0.03, 93.77) | 3.01 (0.05, 196.07) |

**Abbreviations:** OR, odds ratio; CI, confidence interval; CTL, control treatment; CsA, cyclosporine A; TAC, tacrolimus; ST, topical corticosteroid; AS, autologous serum; PRP, platelet-rich plasma; LAC, Lacripep; DQS, diquafosol sodium; CsA+DQS, combined cyclosporine A and diquafosol sodium treatment.
